# Nucleolar-based *Dux* repression is essential for embryonic two-cell stage exit

**DOI:** 10.1101/gad.349172.121

**Published:** 2022-03-01

**Authors:** Sheila Q. Xie, Bryony J. Leeke, Chad Whilding, Ryan T. Wagner, Ferran Garcia-Llagostera, YiXuan Low, Paul Chammas, Nathan T.-F. Cheung, Dirk Dormann, Michael T. McManus, Michelle Percharde

**Affiliations:** 1 MRC London Institute of Medical Sciences, London W12 0NN, United Kingdom;; 2 Institute of Clinical Sciences, Imperial College London, London W12 0NN, United Kingdom;; 3 University of California at San Francisco, San Francisco, California 91413, USA

**Keywords:** 2C-like state, Dux, MERVL, nucleolus, totipotency

## Abstract

Upon fertilization, the mammalian embryo must switch from dependence on maternal transcripts to transcribing its own genome, and in mice this involves the transient up-regulation of MERVL transposons and MERVL-driven genes at the two-cell stage. The mechanisms and requirement for MERVL and two-cell (2C) gene up-regulation are poorly understood. Moreover, this MERVL-driven transcriptional program must be rapidly shut off to allow two-cell exit and developmental progression. Here, we report that robust ribosomal RNA (rRNA) synthesis and nucleolar maturation are essential for exit from the 2C state. 2C-like cells and two-cell embryos show similar immature nucleoli with altered structure and reduced rRNA output. We reveal that nucleolar disruption via blocking RNA polymerase I activity or preventing nucleolar phase separation enhances conversion to a 2C-like state in embryonic stem cells (ESCs) by detachment of the MERVL activator *Dux* from the nucleolar surface. In embryos, nucleolar disruption prevents proper nucleolar maturation and *Dux* silencing and leads to two- to four-cell arrest. Our findings reveal an intriguing link between rRNA synthesis, nucleolar maturation, and gene repression during early development.

## Abstract (precis)

In this study, Xie et al. investigated the mechanisms and requirement for MERVL and two-cell (2C) gene up-regulation in mammalian embryos, and report that robust ribosomal RNA (rRNA) synthesis and nucleolar maturation are essential for exit from the 2C state. Their findings reveal an intriguing link between rRNA synthesis, nucleolar maturation, and gene repression during early development.

Upon fertilization, one of the earliest requirements in the development of a new organism is the formation of a totipotent zygote, which possesses the capacity to generate the entire embryo and all extraembryonic structures. In mice, only the zygote and two-cell stage embryo possess totipotency (Tarkowski 1959; Casser et al. 2017), with subsequent cleavages entailing a decrease in cellular plasticity as cells become specialized. Cells of the E4.5 epiblast, for example, are pluripotent, possessing the ability to generate all three germ layers of the embryo yet typically not extraembryonic cell types (Rossant et al. 2003; Martinez Arias et al. 2013). Concurrent with the establishment of totipotency is the essential switch from reliance on maternal transcripts to activation of the embryo's own genome, termed zygotic or embryonic genome activation (ZGA/EGA). Interestingly, ZGA and totipotency at the two-cell stage have been linked to the rapid and transient activation of several families of transposable elements (TEs), most notably MERVL (Peaston et al. 2004; Svoboda et al. 2004; Macfarlan et al. 2012).

TEs have contributed a widespread and significant source of *cis*-regulatory information to mammalian genomes, providing transcription factor binding sites, enhancers, and promoter sequences (Kunarso et al. 2010; Chuong et al. 2013; Sundaram et al. 2014). Many two-cell-specific and ZGA transcripts use MERVL LTR sequences as promoters, making the MERVL-dependent transcriptome an important component of ZGA (Macfarlan et al. 2011, 2012). In humans, specific TEs from the HERVL family are also expressed upon EGA at the four- to eight-cell stage (De Iaco et al. 2017; Hendrickson et al. 2017). Several studies suggest that correct MERVL regulation is functionally important during embryogenesis. MERVL depletion impairs developmental progression (Huang et al. 2017), while overexpression in embryonic stem cells (ESCs) confers expanded fate potential: the ability in chimeras to generate both embryonic and extraembryonic lineages, similar to two-cell blastomeres (Yang et al. 2020). However, the functional relevance of these TEs at ZGA, as well how and why they are swiftly repressed, is still poorly understood.

Understanding of the two-cell stage and ZGA has been enhanced by the identification of a rare, transient population of cells within ESC cultures that share several epigenetic, metabolic, and transcriptomic features with two-cell embryos, termed two-cell (2C)-like cells (Macfarlan et al. 2012; Bošković et al. 2014), marked by expression of a MERVL-GFP (2C-GFP) reporter. This tool recently led to the discovery of Dux (DUX4 in humans) as a potent MERVL/HERVL and 2C activator. Dux binding directly to 2C/MERVL promoters is sufficient to convert ESCs to a 2C-like fate, and in zygotes and early two-cell embryos it drives the expression of many early ZGA and 2C-specific genes (De Iaco et al. 2017; Hendrickson et al. 2017; Whiddon et al. 2017). Since then, several 2C activators both upstream of and downstream from *Dux* have been uncovered, including both transcriptional and post-transcriptional regulators (Choi et al. 2017; Guallar et al. 2018; Eckersley-Maslin et al. 2019; Hu et al. 2020).

Surprisingly, *Dux* knockout in embryos has overall mild effects, implying the existence of parallel and redundant mechanisms to activate MERVL and ZGA in vivo that remain to be discovered (Chen and Zhang 2019; Guo et al. 2019; De Iaco et al. 2020; Bosnakovski et al. 2021). In contrast, the swift attenuation of *Dux* and MERVL expression for two-cell stage exit is likely essential both in vitro and in vivo. Dux overexpression arrests embryos at the two- to four-cell stage (Guo et al. 2019), while prolonged *Dux* overexpression in ESCs causes DNA damage and apoptosis (Olbrich et al. 2021). Similarly, DUX4 derepression in muscle cells causes the human disease facioscapulohumeral muscular dystrophy (FSHD), characterized by up-regulation of DUX4 target genes, dsRNAs, TEs, and apoptosis (Dixit et al. 2007; Geng et al. 2012; Shadle et al. 2017). Despite its importance, the mechanisms for such rapid shutdown of *Dux* and MERVL gene expression at the late two-cell stage are unclear.

Toward this, we recently reported a novel complex that is essential for *Dux* and MERVL/2C repression during early development, comprising the TE LINE1 in association with nucleolin (Ncl) and Kap1/Trim28 proteins (Percharde et al. 2018). LINE1 RNA in this complex is essential for proper *Dux* repression, and its depletion induces the conversion of ESCs to the 2C-like state and causes two-cell arrest in embryos (Percharde et al. 2018). At the same time, the discovery of Ncl as a *Dux* repressor implied an intriguing potential role for the nucleolus in two-cell exit, which has not yet been explored.

Here, we investigated the impact of nucleolar dynamics and its link to *Dux* repression and two-cell exit using a new 2C-GFP reporter cell system and early mouse embryos. We found that 2C-like cells possess immature nucleoli with morphology akin to nucleolar precursor bodies (NPBs) that show reduced output and abrogated *Dux* repression compared with ESCs. Direct disruption of nucleolar structure and function by RNA polymerase I inhibition (iPol I) or by perturbation of nucleolar liquid–liquid phase separation is sufficient to rapidly release *Dux* from perinucleolar regions, activate its expression, and convert ESCs into a 2C-like state. In vivo, short-term iPol I prevents the formation of mature, Ncl-positive nucleoli from NPBs, activates *Dux*, and impairs developmental progression past the two- to four-cell stage. Our study reveals a direct link between rRNA transcription, nucleolar-based repression, and cell fate during early mammalian development.

## Results

### The 2C-GFP/CD4 reporter enables rapid isolation of endogenous 2C-like cells

**Figure 1.**
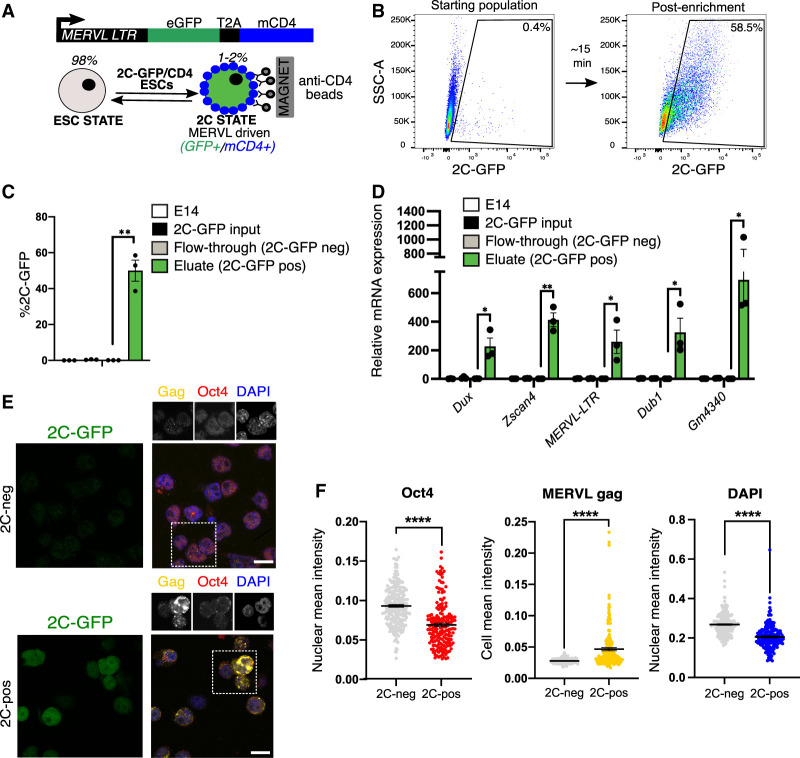
A new reporter cell line for purification of 2C-like cells. (*A*) Reporter design: A previous MERVL-GFP reporter (Ishiuchi et al. 2015) was modified to contain the extracellular portion of the CD4 antigen downstream from GFP and a T2A cleavage element, allowing rapid 2C-like cell purification by anti-CD4 beads. (*B*) Representative flow cytometry plot depicting proportion of typical 2C-GFP^+^ enriched cells (>60% pure, 2C-pos) cells before (*left*) and after (*right*) CD4-based 2C enrichment. (*C*) Percent recovery of 2C-GFP-pos cells after CD4-based purification, comparing CD4^+^ cells (eluate) and CD4^−^ cells (flowthrough). Data are mean ± SEM of three experiments. (*D*) qRT-PCR validation of high levels of 2C-specific genes and MERVL in the 2C-pos, CD4^+^ eluate compared with 2C-neg, CD4^−^ fraction and the starting population. Flowthrough cells are set to 1. Data are mean ± SEM of three experiments. (*E*) Representative confocal images and (*F*) quantification of levels of Oct4 and MERVL gag proteins and DAPI in 2C-pos versus 2C-neg cells following CD4-based purification. Scale bar, 20 µm. All *P*-values represent two-tailed, unpaired Student's *t*-test, with multiple comparisons correction where relevant.

Two-cell (2C)-like cells can be identified from within ESC cultures by expression of a stably integrated fluorescent reporter (e.g., MERVL-GFP, 2C-GFP) (Macfarlan et al. 2012; Ishiuchi et al. 2015). These cells arise infrequently and transiently at a typical rate of <1%–2%, making it challenging to perform large-scale or unbiased analyses in spontaneously arising cells. Purification by flow cytometry assisted cell sorting (FACS) is laborious and slow, thus potentially perturbing biological processes (Binek et al. 2019). To perform 2C-like cell characterisation without flow sorting, we devised an improved strategy to allow FACS-free and rapid isolation of 2C-like cells. We generated ESCs stably harbouring a modified MERVL-GFP reporter, which induces expression of the extracellular portion of CD4 protein as well as GFP in the 2C state (2C-GFP/CD4^+^) (Fig. 1A). With this technique, naturally arising 2C-like cells can be rapidly purified from ESC cultures by magnetic bead-based isolation with a typical purity of 55%–85% after only 15 min, more than a 100-fold increase over the starting population (Fig. 1B,C; Supplemental Fig. S1A,B). We confirmed that 2C-GFP/CD4^+^-enriched cells (“2C-pos”) express markers of bona fide 2C-like cells, including high levels of MERVL and 2C-specific transcripts (Fig. 1D; Supplemental Fig. S1C), similar to FACS-purified 2C-like cells (Percharde et al. 2018). 2C-GFP/CD4^+^ cells display induction of MERVL Gag protein, together with loss of Oct4 protein and DAPI-dense chromocenters (Fig. 1E,F), all of which are previously described features of 2C-like cells and similar to two-cell embryos (Macfarlan et al. 2012; Ishiuchi et al. 2015; Percharde et al. 2018). Thus, 2C-GFP/CD4^+^ cells faithfully capitulate the 2C-like state.

### 2C-like cell nucleoli resemble NPBs and exhibit reduced nucleolar function

**Figure 2.**
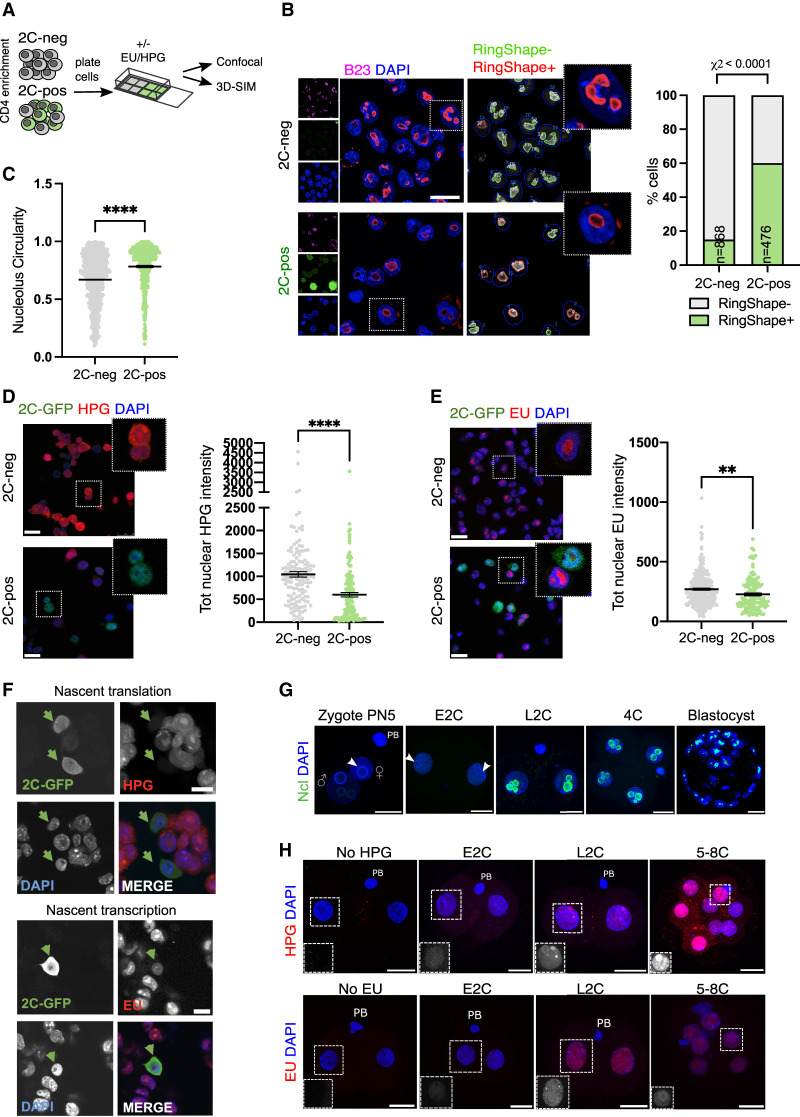
2C-like cells and embryos have altered nucleolar morphology and function. (*A*) Experimental set-up for 2C-like cell profiling: Following CD4-based enrichment, 2C-neg/pos populations were plated into Matrigel-coated chambers for a minimum of 1 h before the indicated downstream applications. (*B*) Immunofluorescence images and quantification (RingShape+, CellProfiler) appearance in 2C-pos/neg cells, revealing that 2C-like cell nucleoli (2C-GFP, green), stained by the nucleolar marker B23 (Npm1, purple), have rounded, ring-like morphology. (*n*) Number of cells scored. Scale bar, 20 µm. (*C*) Nucleolar circularity is significantly increased in 2C-like cells. Very small nucleoli (area <100 pixels) were filtered out as can typically generate unreliable measurements (see the Materials and Methods). (*D*,*E*) Immunofluorescence images and quantification of nascent translation (*D*) and nascent transcription (*E*) rates in 2C-pos versus 2C-neg cells via HPG or EU Click-iT incorporation experiments, respectively. Scale bar, 25 µm. (*F*) Confirmation of reduced transcription and translation in 2C-like (2C-GFP^+^) cells within unsorted populations, using an independent 2C-GFP cell line (Percharde et al. 2018). Scale bar, 10 µm. (*G*) Nucleolar (nucleolin [Ncl]) staining in in vitro cultured embryos, showing the emergence of Ncl^+^ nucleoli at the late two-cell stage (L2C). (E2C) Early two-cell stage, (PB) polar body. White arrows denote NPBs. Scale bar, 20 µm. (*H*) Analysis of nascent transcription/translation in embryos by EU/HPG assays, respectively. Scale bar, 20 µm. *Insets* show EU/HPG staining alone (grayscale) in a representative blastomere from each image. *P*-values represent χ^2^ test (*B*) and two-tailed Student's *t*-test (*C*–*E*), with Welch's correction for uneven variance where relevant; data represent at least two independent experiments.

We previously discovered that a ribonucleoprotein complex comprising LINE1 RNA, together with Nucleolin (Ncl) and Kap1, is essential for both ribosomal RNA (rRNA) expression as well as 2-cell exit (Percharde et al. 2018). Since Ncl and rRNA are both well-known nucleolar components, we investigated whether the 2C-like state is associated with changes to nucleoli. 2C-positive and negative cells were isolated following CD4 enrichment and examined by confocal microscopy (Fig. 2A). Interestingly, we found that 2C-like cells possess a distinct nucleolar morphology, with a rounded, ring-like structure (Fig. 2B,C). We next tested whether nucleolar morphological changes in 2C-like cells might also be accompanied by changes to RNA Polymerase I (Pol I) activity and nucleolar function. Nucleoli are the site of Pol I-driven ribosomal RNA (rRNA) synthesis, processing, and ribosomal assembly. rRNA makes up >70% of cellular RNA, which is tightly coordinated with *Rpl/Rps* RNA expression and protein synthesis (Laferté et al. 2006; Percharde et al. 2017). We measured production of nascent RNA and protein in the 2C-like versus ESC state using Click-iT assays, where a pulse of nucleotides or amino acid analogues is given to cells that are then fluorescently labeled after fixation for quantification (Fig. 2A). We discovered a significant reduction in translation in 2C-like cells (Fig. 2D) as well as reduced nascent RNA synthesis, the majority of which comprises nucleolar rRNA (Fig. 2E; Supplemental Fig. S2A,B, inset). To confirm that these changes are not an artefact of CD4-based enrichment, nascent transcription and translation rates were profiled in unsorted, bulk 2C-GFP reporter ESCs (Percharde et al. 2018). In agreement, spontaneously arising 2C-like cells exhibit reductions in nucleolar function in contrast to neighboring ESCs (Fig. 2F; Supplemental Fig. S2B).

Subsequently, we investigated whether these changes are reflected at the two-cell stage in vivo. Following fertilization, one- to two-cell embryos possess immature nucleolar precursor bodies (NPBs)—largely uncharacterized structures that are initially transcriptionally silent and lacking distinct compartments (Fléchon and Kopecny 1998). In contrast, mature nucleoli contain three subcompartments: a fibrillar center surrounded by a dense fibrillar component, which itself is surrounded by a granular component. In contrast to mature nucleoli, embryo NPBs are large, circular, and morphologically similar to 2C-like nucleoli, except they lack strong staining for Ncl (Fig. 2B,G, white arrows). Coincident with the increasing initiation of rRNA transcription, mature nucleoli only gradually form from NPBs at the late two-cell stage onward (Kyogoku et al. 2014; Borsos and Torres-Padilla 2016). We analyzed nucleolar function in embryos with nascent transcription/translation assays, which demonstrated dynamic rates of biosynthesis during the two-cell stage. Early two-cell (E2C) embryos exhibit low levels of nascent RNA synthesis but also nucleolar translation, which rapidly increases by the late 2C (L2C) stage and upon two-cell exit (Fig. 2H). At the same time, Ncl protein only becomes readily detectable surrounding nucleoli in L2C embryos onward (Fig. 2G), at the time when MERVL and the two-cell program is being shut down. We conclude that the two-cell stage in vitro and in vivo is characterized by the presence of immature NPBs with significantly reduced nucleolar function and morphologically distinct nucleolar structure.

### Nucleolar disruption induces conversion to the 2C-like state

**Figure 3.**
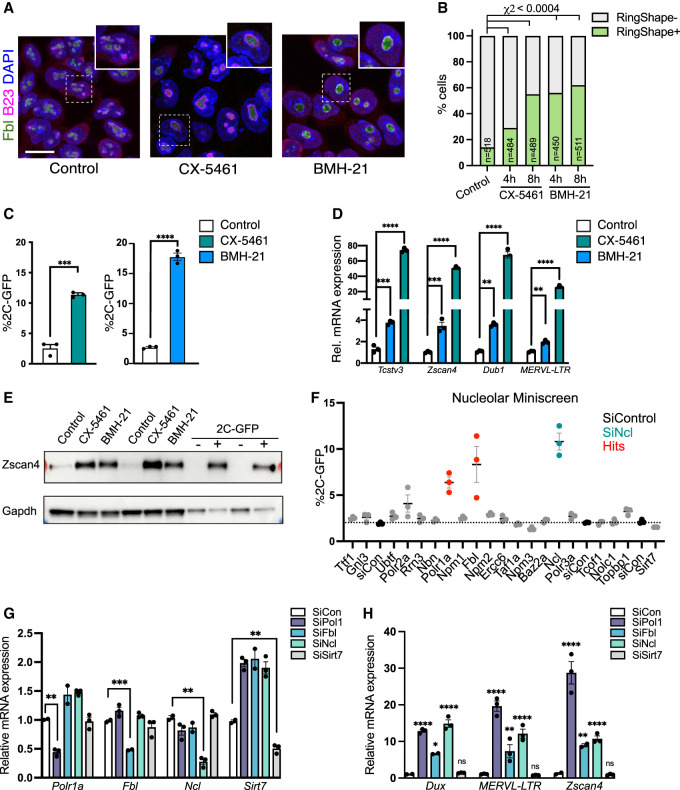
Nucleolar disruption induces the 2C-like state. (*A*) Representative immunofluorescence images following staining for nucleolar markers (fibrillarin [Fbl]) and B23 4 h after RNA Pol I inhibition (iPol I) with either CX-5461 or BMH-21. Scale bar, 20 µm. (*B*) Quantification of the percentage of cells with ring-like (RingShape+) nucleoli 4 and 8 h after iPol I. *P*-values, χ^2^ test adjusted for multiple comparisons. (*n*) Number of cells. (*C*) Percentage of 2C-GFP^+^ cells following overnight (16- to 24-h) treatment with 0.25 µM iPol I. Data are mean ± SEM. *n* = 3 biological replicates representative of three or more experiments. (*D*) qRT-PCR analysis of 2C-specific genes and TEs following iPol I as in C, with *P*-values in *C* and *D* representing two-tailed *t*-test with two-stage multiple comparisons correction. (*E*) Western blots showing up-regulation of 2C-specific protein Zscan4 after 16- to 24-h iPol I in ESCs, shown next to purified 2C-GFP/CD4^+/−^ cells. Replicates from two experiments are shown. (*F*) Flow cytometry analysis of percentage of 2C-GFP^+^ cells following siRNA knockdown of the indicated factors. Red samples indicate a *Z*-score of >1, with KD of Ncl shown as a positive control (teal). Data are mean ± SEM of three biological replicates, representative of two repeats of the screen. (*G*,*H*) Validation by qRT-PCR of siRNA-mediated knockdown of the indicated factors (*G*) and up-regulation of 2C-specific genes (*H*) showing mean ± SEM of *n* = 2–3 biological replicates, representative of two experiments. *P*-values, two-way ANOVA followed by Dunnett's multiple comparisons test.

The observed nucleolar remodeling upon two-cell stage exit led us to ask whether alterations to nucleolar structure and function might drive the 2C-like state. We took advantage of two different small molecules to inhibit RNA Pol I and rRNA synthesis: CX-5461, which blocks recruitment of the Pol I initiation factor SL1 to rDNA (Bywater et al. 2012; Haddach et al. 2012), and BMH-21, which triggers rapid Pol I degradation (Peltonen et al. 2014). We found that nucleolar disruption by mild or partial inhibition of rRNA synthesis (Pol I inhibition [iPol I]) is detectable by 2 h (Supplemental Fig. S3A), and by 4 h induces morphological nucleolar remodeling, generating singular ring-like structures in ESCs resembling 2C-like nucleoli and embryo NPBs (Figs. 2G, 3A,B). Importantly, the structures observed following this milder inhibition are distinct from the nucleolar cap-like structures seen upon more extreme nucleolar stress, where fibrillar proteins such as Fibrillarin (Fbl) or UBF aggregate at the nucleolar periphery, or from complete dissolution of nucleolar proteins into the nucleoplasm (Supplemental Fig. S3B; (Shav-Tal et al. 2005; Ide et al. 2020). Moreover, we did not detect gross changes to Ncl or Fbl protein abundance upon iPol I (Supplemental Fig. S3C,D). Strikingly, we found that following overnight nucleolar reprogramming, iPol I causes a significant increase to 20% of 2C-like cells within ESC cultures (Fig. 3C). In agreement, iPol I induces high expression of 2C-specific genes and MERVL transcripts (Fig. 3D) as well as MERVL gag protein (Supplemental Fig. S3E). The 2C-specific protein Zscan4 is also highly elevated following iPol I treatment, compared with purified 2C-GFP/CD4^+^ cells as a positive control (Fig. 3E). Thus, nucleolar disruption produces 2C-like NPBs and moreover is sufficient to reprogram ESCs into the 2C-like state.

### Nucleolar proteins driving rRNA synthesis and processing are essential for *Dux* and 2C repression

These results support the hypothesis that the development of functionally mature nucleoli may play a role in the repression of the two-cell transcriptional program and exit from the two-cell stage. To investigate this, we asked which nucleolar proteins are most important for repression of the 2C-like state in ESCs and performed an siRNA miniscreen for nucleolar components in 2C-GFP ESCs. Similar to the effects of Ncl loss, we found that depletion of RNA Pol I and Fbl also causes a notable increase in 2C-like cells (Fig. 3F; Supplemental Fig. S3F). Knockdown (KD) of other nucleolar proteins such as Sirt7 has a limited effect, suggesting a reliance on specific factors for repression of the 2C-like state. Conversely, KD of Npm3, a negative regulator of ribosome biogenesis (Huang et al. 2005), led to a small but consistent reduction in 2C-GFP^+^ cells (Fig. 3F). Confirming these results, siRNAs against Pol I, Fbl, and Ncl all induce high levels of 2C-specific genes and transposons (Fig. 3G,H), indicating that these factors are necessary for repression of the 2C-like state. Interestingly, RNA Pol I, Ncl, and Fbl are all known to be critical for rRNA synthesis and/or processing (Ginisty et al. 1998; Yao et al. 2019; Ide et al. 2020). In contrast, knockdown of nonnucleolar RNA Pol III (Jiang et al. 2020), which transcribes small RNAs, tRNAs, and 5S rRNA, caused no activation of the 2C-like state (Fig. 3F; Supplemental Fig. S3G). Collectively, these data reveal an intriguing link between nucleolar rRNA synthesis and 2C repression to maintain ESC identity.

**Figure 4.**
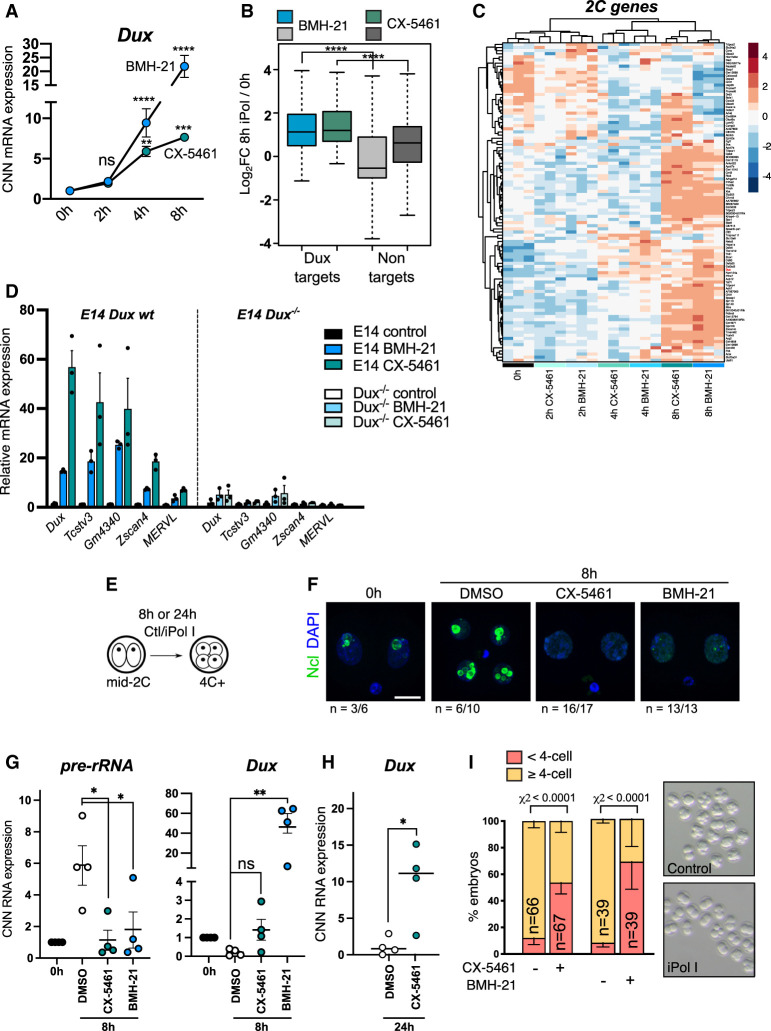
Nucleolar disruption causes *Dux* reactivation in ESCs and embryos. (*A*) Cell number-normalized (CNN) qRT-PCR time-course analysis of *Dux* up-regulation following iPol I. Data were analyzed as CNN to exclude potential global effects of iPol I on transcription; however, the same results are seen with *Rpl7/H2A* normalization. Data are mean ± SEM. *n* = 3 biological replicates. *P*-values, two-way ANOVA and Šídák multiple comparisons test. (*B*) Box plot of log_2_ fold change values for *n* = 99 *Dux* target genes (Percharde et al. 2018) versus significantly altered nontargets (FDR < 0.05, CX-5461: *n* = 8057; BMH-21: *n* = 13,830) following 8-h iPol I. *P*-values, two-sided Wilcoxon rank sum test. (*C*) Heat map of 2C-specific genes (Macfarlan et al. 2012) showing gradual up-regulation following iPol I. Samples are grouped by unsupervised hierarchical clustering. (*D*) Expression of *Dux* and 2C-specific genes in wild-type versus *Dux*^*−/−*^ E14 ESCs. The control for each cell line is set to 1. Data are mean ± SEM of three biological replicates, representative of two experiments. (*E*) Schematic for embryo iPol I inhibitor experiments with 1 µM BMH-21 or CX-5461. (*F*) Ncl immunofluorescence in mid-2C embryos fixed immediately or cultured for 8 h with the indicated inhibitors. (*n*) Number of embryos with the representative staining from two experiments. Scale bar, 20 µm. (*G*) CNN qRT-PCR expression data following 8-h iPol I in mid two-cell embryos showing inhibited *Dux* repression. Data are mean ± SEM. *n* = 4 experiments with equal numbers of embryos, with levels at 0 h set to 1 in each experiment. *P*-values, one-way ANOVA with Dunnett multiple comparisons correction. (*H*) CNN qRT-PCR expression data showing *Dux* up-regulation after 24 h for 1 µM CX-5461. Data are mean ± SEM. *n* = 4 experiments. *P*-values, Welch's two-tailed *t*-test. (*I*) Embryo progression rates following 24-h iPol I treatment in *n* = 4 experiments (CX-5461) and *n* = 2 experiments (BMH-21). *P*-values, χ^2^ test. *n* = number of embryos.

Next, we asked how rRNA synthesis and nucleolar function are mechanistically linked to repression of the 2C-like state. We focused on Dux, which is a potent MERVL and 2C-like gene activator and is up-regulated upon nucleolar protein knockdown (Fig. 3H). We performed time-course experiments of acute iPol I treatment followed by qRT-PCR and RNA-seq (Fig. 4A; Supplemental Fig. S4A) and found that *Dux* is significantly induced as early as 4 h following nucleolar disruption (Fig. 4A; Supplemental Fig. S4B). By 8 h, Dux targets are highly up-regulated among all significantly altered genes following iPol I (Fig. 4B). Moreover, transcriptomic profiling of 2C-specific genes (Macfarlan et al. 2012; Percharde et al. 2018) demonstrated that MERVL and the 2C program are widely up-regulated following nucleolar disruption (Fig. 4C; Supplemental Fig. S4B,C). Next, to test whether 2C-like gene induction is dependent on Dux, we performed iPol I experiments in *Dux*^*−/−*^ ESCs (Grow et al. 2021) compared with wild-type (wt) E14 ESCs. 2C genes are highly up-regulated by iPol I, which is fully prevented upon *Dux* deletion (Fig. 4D; Supplemental Fig. S4D). We subsequently investigated whether iPol I can also prevent nucleolar maturation and *Dux* silencing in mid-two-cell embryos (Fig. 4E), which normally occur rapidly as embryos transit to the late two-cell stage (De Iaco et al. 2017; Hendrickson et al. 2017). We found that iPol I treatment in mid-2C embryos prevents the maturation of Ncl-positive nucleoli from NPBs (Fig. 3F; Supplemental Fig. S4E,F) and causes significant rRNA reduction and concomitant *Dux* activation in L2C–4C embryos (Fig. 4G,H). We observed slightly different kinetics with the two inhibitors, with BMH-21 causing more rapid activation of *Dux* in embryos than CX-5461 (Fig. 4G,H), similar to in ESCs (Fig. 4A). Finally, nucleolar disruption leads to an inability to progress beyond the two- to four-cell stage, in contrast to control embryos (Fig. 4I; Supplemental Fig. S4G). Together, these results indicate that nucleolar disruption rapidly leads to *Dux* derepression and induction of the two-cell state.

### *Dux* is repressed in perinucleolar chromatin

Prolonged treatment or high doses of drugs that perturb rRNA synthesis or cause rDNA damage is known to activate nucleolar stress. In this process, disruption of nucleolar integrity releases ribosomal proteins into the nucleoplasm to bind MDM2, leading to p53 stabilization and activation followed by downstream effects such as cell cycle arrest (Rubbi and Milner 2003; James et al. 2014). To understand how nucleolar disruption is linked to *Dux* activation, we first tested whether this is dependent on nucleolar stress. Although iPol I does not induce typical markers of nucleolar stress (Fig. 3A; Supplemental Fig. S3B), levels of total and activated p53 (phospho-p53) are increased upon iPol I, similar to the effect of the topoisomerase II inhibitor etoposide, used as a positive control (Supplemental Fig. S5A). Interestingly, etoposide treatment also increases the proportion of 2C-GFP^+^ cells in culture (Supplemental Fig. S5B), suggesting that p53 activation can activate the 2C-like state. Indeed, a recent study reported DNA damage-dependent activation of Dux/DUX4 and the 2C-like state via p53 (Grow et al. 2021). However, we did not detect any increase in phospho-p53 in endogenously arising 2C-like cells (Supplemental Fig. S5C). Furthermore, iPol I treatment is still able to cause significant *Dux* activation in the absence of p53 (Supplemental Fig. S5D; Bowling et al. 2018). Thus, although p53 activation is sufficient to induce the 2C-like state upon DNA damage (Grow et al. 2021), it is not strictly necessary for *Dux* derepression upon nucleolar disruption.

**Figure 5.**
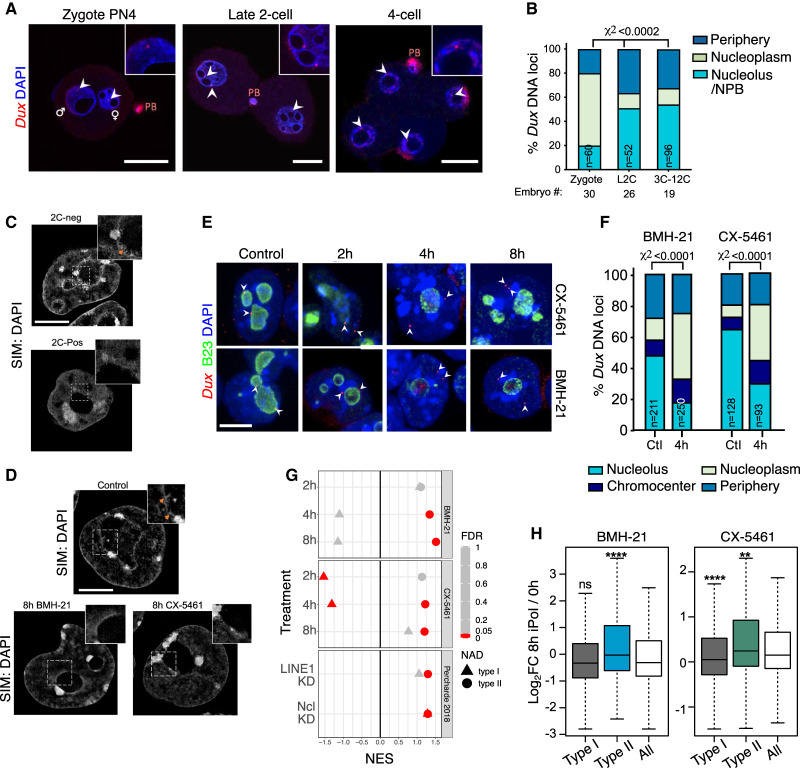
Nucleolar disruption induces *Dux* relocalization and activation. (*A*,*B*) Representative confocal images and scoring of *Dux* localization in the indicated embryo stages. (*n*) Number of pronuclei or nuclei; embryos from two independent experiments were scored. *P*-values, χ^2^ test. Scale bar, 20 µm. (*C*,*D*) Example images of chromatin distribution as marked by DAPI staining in 3D-SIM imaging experiments in 2C-pos versus 2C-neg cells (*C*) and in ESCs upon 8-h iPol I (*D*). (*D*) 2C-neg cells and control but not iPol I ESCs have nucleolar chromatin fibers, visible as a roughened nucleolar border (orange arrows, *inset*). Scale bar, 5 µm. (*E*) Representative immuno-DNA FISH images at the indicated time points of iPol I for *Dux* alleles (red) compared with nucleolar (B23, green) or nuclear lamina (LaminB; not shown) compartments. Scale bar, 10 µm. (*F*) Quantification of *Dux* localization at 4-h iPol I showing movement away from the nucleolus. *P*-values, χ^2^ test. (*n*) Number of nuclei scored. (*G*) Dot plot of GSEA enrichment scores (NES) and significance (FDR) for type I or type II NADs using expression data following iPol I or following LINE1/Ncl KD (Percharde et al. 2018). (*H*) Box plot of log_2_ fold change values for type I NADs (*n* = 1565) or type II NADs (*n* = 371) versus all genes at 8-h iPol I. *P*-values, two-sided Wilcoxon rank-sum test, comparing type I/II NADs with all genes.

In ESCs but not 2C-like cells, we previously reported that the *Dux* genes localize to perinucleolar regions with unknown functional relevance (Percharde et al. 2018). We next tested the hypothesis that this is mirrored in vivo upon nucleolar maturation and may thus be linked to the functional regulation of *Dux*. Embryo DNA FISH revealed that *Dux* loci are indeed recruited to nucleoli in late two-cell embryos onward but not to zygote NPBs (Fig. 5A,B). These data indicate that *Dux* recruitment to the nucleolar periphery is a hallmark of two-cell exit. Mature nucleoli are surrounded by a shell of chromatin that is enriched for repressive histone marks (Németh et al. 2010; Lu et al. 2020) and is lowly transcribed (Quinodoz et al. 2018). We therefore reasoned that disruption of nucleolar function and morphology might lead to *Dux* up-regulation by preventing its repression at the nucleolar periphery. To observe perinucleolar chromatin in more detail, we performed 3D superresolution structured illumination microscopy (3D-SIM) of DAPI staining in ESCs versus 2C-like cells. These experiments revealed a reduction in perinucleolar chromatin fibers in the 2C-like state (Fig. 5C, orange arrows), alongside a previously reported loss of chromocenters (Ishiuchi et al. 2015). Reduced nucleolar DNA association is moreover replicated by 8 h of BMH-21 and CX-5461 treatment (Fig. 5D), suggesting that iPol I rapidly perturbs nucleolar-associated chromatin. We next examined whether nucleolar disruption alters the localization of the *Dux* gene locus, focusing on acute inhibition to determine direct effects of iPol I. DNA FISH confirmed that *Dux* is frequently associated with perinucleolar regions in ESCs, and moreover revealed significant movement away from nucleoli to the nucleoplasm by 4 h of either CX-5461 or BMH-21 (Fig. 5E,F). Using 3D nuclear segmentation and analysis of *Dux* distance to nuclear compartments, we confirmed these findings and found that movement away from the nucleolus starts from 2-h CX-5461 and is detected robustly at 4 h. In contrast, there is no change in *Dux* distance from the lamina (Supplemental Fig. S6A–C). Thus, it is only nucleolar-localized *Dux* alleles that are affected by iPol I. Subsequently, we used RNA FISH to monitor the appearance of *Dux* mRNA in single cells and found that *Dux* movement is closely linked to reactivation of *Dux* RNA expression (Supplemental Fig. S7A). We next scored the location of putative nascent RNA foci (scored as RNA in nuclei with two or fewer foci, and the two brightest spots in nuclei with multiple foci) (Supplemental Fig. S7B; Mueller et al. 2013). Importantly, nascent *Dux* expression occurs only in the nucleoplasmic compartments, in strong agreement with the repressive nature of the nucleolus (Supplemental Fig. S7C).

Nucleolar-associated DNA regions (NADs) have been previously identified by isolation and sequencing of DNA associated with purified nucleoli (NAD-seq) (Németh et al. 2010; Vertii et al. 2019), which has confirmed the generally repressive nature of nucleolar chromatin (Lu et al. 2020). Using NAD annotations generated from ESCs (Bizhanova et al. 2020), we asked whether the expression of other nucleolar-associated genes is altered following nucleolar disruption. We looked at type I NADs, which overlap constitutively lamina-associated domains (cLADs) and are considered to comprise constitutive heterochromatin, and type II NADs, which do not overlap LADs in multiple cell types (Peric-Hupkes et al. 2010). GSEA revealed that type II NAD genes are particularly sensitive to nucleolar disruption and are significantly up-regulated from 4-h iPol I compared with all genes, in contrast to type I NADs (Fig. 5G,H). This is not an isolated effect of inhibitor treatment, as knockdown of Ncl or LINE1, both important for nucleolar function (Percharde et al. 2018; Lu et al. 2020), also led to NAD and NAD/LAD gene up-regulation (Fig. 5G). Together, these results reveal that nucleolar association of *Dux* is closely tied to its repression and suggest that iPol I induces global disruption of nucleolar chromatin organization and gene expression.

### Phase-separated nucleolar integrity is required for *Dux* repression at nucleoli

**Figure 6.**
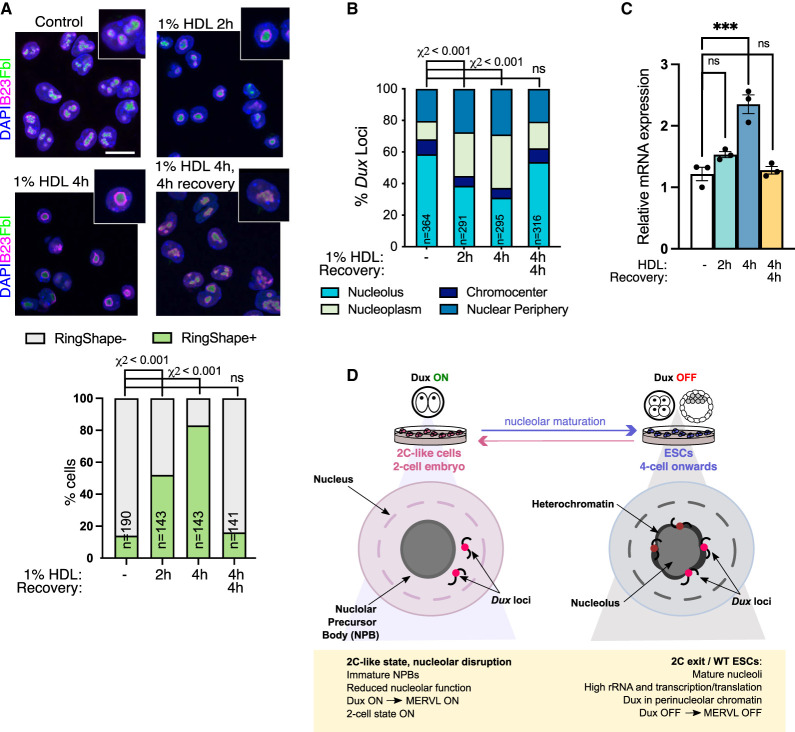
Disruption of LLPS induces *Dux* movement and activation. (*A*) Immunofluorescence for nucleolar markers B23 and Fbl after the indicated times of incubation with 1% 1,6-hexanediol (HDL) with or without washout and recovery in normal media, and quantification of the percentage of cells with RingShape+ nucleoli (*below*). Scale bar, 20 µm. (*n*) Number of cells. *P*-values, χ^2^ test with Bonferroni adjustment for multiple comparisons. (*B*) Scoring of *Dux* locus nuclear positioning following HDL treatments from *Dux* immuno-FISH experiments. (*n*) Number of nuclei scored from two FISH experiments. *P*-values, χ^2^ test, with Bonferroni adjustment for multiple comparisons. (*C*) Expression of *Dux* by qRT-PCR following HDL treatment. Data are mean ± SEM for *n* = 3 biological replicates, representative of two independent experiments. *P*-values, one-way ANOVA with Dunnett correction for multiple comparisons. (*D*) Model: Nucleolar maturation allows for Dux repression and two-cell exit. In early embryos and 2C-like cells, NPBs have altered morphology, reduced function, and reduced chromatin association. We propose that this provides a permissive environment for *Dux* and subsequent 2C/MERVL expression. In mature nucleoli with high rRNA output, *Dux* is recruited to perinucleolar chromatin and is repressed. Disruption of nucleolar integrity via iPol I or inhibition of nucleolar phase separation releases *Dux* and leads to its derepression.

Last, we sought to determine the link between disrupted rRNA synthesis and *Dux* locus release and derepression. The membrane-less nucleolus is held together by liquid–liquid phase separation (LLPS), which is driven by the association of rDNA with nucleolar proteins and moreover is dependent on continual rRNA synthesis (Feric et al. 2016; Yao et al. 2019; Ide et al. 2020). We hypothesized that the disruption of rRNA synthesis may inhibit nucleolar integrity and LLPS, thus allowing the release of associated DNA regions such as *Dux*. To test this, we used 1,6-hexanediol (HDL), an aliphatic alcohol used to disrupt liquid-like condensates (Ribbeck and Gorlich 2002). Short-term treatment with 1% HDL—a dose notably lower than typically used to disrupt nonnucleolar compartments (Vertii et al. 2019)—is sufficient to alter nucleolar morphology, resembling 2C-like cells or iPol I treatment (Fig. 6A). Disruption of phase separation remarkably releases *Dux* loci after only 2-h HDL (Fig. 6B), and moreover leads to significant *Dux* up-regulation by 4 h (Fig. 6C). Furthermore, we found this to be highly dynamic, with nucleolar morphology, *Dux* localization, and transcriptional repression all returning to normal after 4-h washout (Fig. 6C). These results suggest that *Dux* localization and repression are maintained in the nucleolus through LLPS. Taken together, our data show that nucleolar disruption by several means causes *Dux* reactivation, initiation of 2C/MERVL gene transcription, and conversion back to the 2C-like state. In vivo, *Dux* loci become recruited to maturing NPBs at the late two-cell stage, and disruption of nucleolar maturation prevents *Dux* repression. Overall, these findings point to a novel requirement for rRNA biogenesis, nucleolar maturation, and nucleolar-based repression for correct cell identity during the earliest stages of embryo development.

## Discussion

Major ZGA is an essential process occurring at the two-cell stage of early mouse embryogenesis, which entails rapid activation of zygotic RNAs required for subsequent development. This includes a significant number of transcripts driven by the TE MERVL, which, unlike other ZGA transcripts, are rapidly down-regulated upon two-cell exit. These dynamics swiftly follow the rapid repression of the MERVL activator, Dux. Sustained Dux expression in two-cell embryos is poorly tolerated and moreover promotes persistence of the 2C program and impedes development (Percharde et al. 2018; Guo et al. 2019). Thus, timely Dux repression is essential, yet the mechanisms for this process are poorly understood.

Here, we reveal that high rRNA synthesis and nucleolar maturation from inactive NPBs are essential drivers of *Dux* repression in embryos and 2C-like cells. The absence of pluripotency proteins such as Oct4 is a well-known feature of the 2C-like state (Macfarlan et al. 2012). Here we place this finding within the context of suppression of both rRNA transcription and global translation and reveal that the 2C-like state is characterized by significantly reduced nucleolar function, akin to NPBs in two-cell embryos. NPBs are unique structures that in one- to two-cell embryos lack distinct compartments and exhibit low rRNA synthesis (Fléchon and Kopecny 1998; Borsos and Torres-Padilla 2016). Nucleolar maturation occurs with the resumption of transcription and is essential to generate high levels of rRNA and promote ribosomal assembly to fuel embryonic growth. However, it is becoming clearer that nucleoli also possess other roles in development. NPBs are essential for early centromeric chromatin organization, which localizes to the surface of NPBs (Zuccotti et al. 2005; Fulka and Langerova 2014), and nucleolus removal in oocytes causes two-cell arrest (Ogushi et al. 2008). Intriguingly, reprogramming to totipotency by somatic cell nuclear transfer (SCNT) generates NPBs after only 3 h (Martin et al. 2006), highlighting a link between totipotency and nucleolar biology. Here, we show that 2C-like cells have NPB-like nucleoli with reduced chromatin association. We propose that nucleolar maturation and full nucleolar function are critical for *Dux* recruitment to the nucleolar periphery for its repression, which in turn is essential for two-cell exit. Conversely, mild inhibition of Pol I is sufficient to rapidly release *Dux* from nucleolar chromatin and to activate its expression (Fig. 6D). In this way, we hypothesize that ZGA itself provides the mechanism to shut down the 2C program in a feedback loop whereby high levels of rRNA synthesis promote nucleolar maturation that can then silence Dux.

Importantly, this mechanism of Dux regulation appears separate from nucleolar stress-mediated p53 activation, which is capable of directly inducing *Dux* (Grow et al. 2021). We found that iPol I ESCs or 2C-like cells do not display typical nucleolar stress markers, and iPol I ESCs still activate *Dux* in the absence of p53, albeit with slightly reduced levels. In contrast, p53 activation upon longer or more severe nucleolar stress in ESCs may also significantly contribute to *Dux* activation (Sun et al. 2021; Yu et al. 2021). Instead, our data on acute, milder nucleolar disruption agree with previous nucleolar gene positioning studies, which demonstrated in yeast that an ectopic rDNA repeat can silence its chromosomal region (Zuccotti et al. 2005) and that 5S rDNA sequences are sufficient to induce nucleolar association and silencing of a reporter gene in ESCs (Fedoriw et al. 2012). Indeed, NAD-seq data (Bizhanova et al. 2020) indicate that *Dux* is located within a NAD (Supplemental Fig. S8). Interestingly, *D4Z4* repeats containing human *DUX4* have also been proposed to reside within a NAD (Németh et al. 2010) and to be bound by NCL (Gabellini et al. 2002). Studying nucleolar regulation of *DUX4* will allow us to understand whether failure of a similar mechanism may contribute to DUX4 derepression in the disease FSHD. It will also be important in future studies to determine whether other aspects of nucleolar biology are important for Dux repression and why particular factors (Ncl, Fbl, and Pol I), but not other nucleolar proteins, are particularly important.

Our data raise the question of how rRNA transcription is tied to *Dux* nucleolar association and repression. The nucleolus is self-organized into its three subdomains by phase separation, driven by the interaction between nucleolar proteins and rDNA (Feric et al. 2016; Yao et al. 2019), and nucleated by rRNA (Falahati et al. 2016). Indeed, purified Fbl and B23 (Npm1) can separate into distinct layers and recapitulate the dense fibrillar component and granular component in solution (Feric et al. 2016). Importantly, the phase separation properties of the nucleolus in cells rely on continual activity of RNA Pol I, since its inhibition leads to disruption of these compartments (Ide et al. 2020). Our results point to a model in which *Dux* is held in repressive perinucleolar heterochromatin that is maintained through LLPS, with perturbation of rRNA transcription or direct inhibition of phase separation sufficient to cause *Dux* dissociation and derepression. Similarly, NPB structures of zygotes and 2C-like cells are not associated with *Dux* loci, and we propose that they are not competent for *Dux* repression. It will be interesting in future work to understand in further depth how the nucleolar periphery provides a repressive compartment like the nuclear lamina (Kind and van Steensel 2010). We previously found that LINE1 RNA and repressors Kap1 and Ncl bind both *Dux* and rDNA in ESCs (Percharde et al. 2018), with a recent report also identifying nucleolar Lin28 as a *Dux* repressor within this complex (Sun et al. 2021). Furthermore, it is likely that other repressor proteins may colocalize at the nucleolar periphery. For example, the histone methyltransferase G9a has been reported in the nucleolus (Yuan et al. 2007), while a repressive role for nucleolar RNA Pol II itself has been recently discovered (Abraham et al. 2020).

In addition to *Dux* regulation, our work points to a wider role for nucleolar chromatin in gene regulation and its dynamic establishment in early embryos. RNA-seq data upon iPol I suggest that genes within type II NADs, regions that are only associated with nucleoli and not constitutive LADs, are most sensitive to nucleolar disruption. In contrast, type I NADs that show both nucleolar and constitutive lamina association are not up-regulated upon acute iPol I, in agreement with their classification as constitutive heterochromatin and their low expression (Vertii et al. 2019; Bizhanova et al. 2020). Toward this, HDL treatment in MEFs was shown to cause relocalization of a type II, but not type I, NAD (Vertii et al. 2019). Future work is needed to understand whether these distinct NAD classes show differences in their association strength with the nucleolus or whether their activation depends on further mechanisms or factors upon dissociation. For example, neuronal NAD genes detach from the nucleolus upon neural progenitor cell differentiation but do not yet become activated, supporting the model that their release might poise them for later expression (Bersaglieri et al. 2020).

More broadly, it will be important in future work to understand how nucleolar chromatin organization proceeds in early embryos, as well as to uncover which genes rely on nucleolar association for their repression. Together with new findings of nucleolar function/dysfunction in multiple processes such as protein quality control (Azkanaz et al. 2019; Frottin et al. 2019), cancer (Lindström et al. 2018), and aging (Buchwalter and Hetzer 2017; Tiku and Antebi 2018), our data reveal a novel axis of nucleolar biology in early development and reflect the multifaceted function of the nucleolus.

## Materials and methods

### Mice and embryos

All animal experiments were performed according to a UK Home Office Project License in a Home Office-designated facility using 4- to 6-wk-old female and 2- to 6-mo-old male CD1 mice (Charles River). Animals were maintained on a 12-h light/dark cycle and provided with ad libitum food and water in individually ventilated cages. Female mice were superovulated by intraperitoneal injection of 5 IU of pregnant mare serum gonadotropin (PMSG; Folligon, MSD Animal Health), followed by 5 IU of human chorionic gonadotropin (hCG; Chorulon, MSD Animal Health) 46–48 h later, and then placed immediately with males. Zygotes were collected from oviducts ∼22–24 h after hCG in M-2 medium (Sigma M7167), isolated from cumulus cells with 200 µg/mL hyalurionidase (Sigma H3506), washed through successive drops of M-2, and then cultured in pre-equilibrated KSOMaa (Sigma MR-106-D) in microdrops overlaid with mineral oil (Sigma M5310) or in four-well dishes. Zygotes were cultured in a humidified incubator at 37°C and 5% CO_2_ until early two-cell (31–33 h after hCG), late two-cell (48–49 h after hCG), morula (3 d after hCG), or blastocyst (4 d after hCG) stage.

### ESC culture

Mouse E14Tg2A (E14) ESCs (male) were used for all experiments (Hooper et al. 1987) and to derive 2C-GFP reporter cells. 2C-GFP reporter ESCs were described previously (Percharde et al. 2018) and were used when prior purification of larger numbers of 2C-like cells was not needed or for validation. All ESCs were cultured at 37°C with 5% CO_2_ on 0.1% gelatin-coated plates in ES-FBS culture medium (high-glucose DMEM GlutaMAX with sodium pyruvate [Thermo Fisher Scientific], 15% FBS [Gibco], 0.1 mM nonessential amino acids [Gibco], 0.1 mM 2-mercaptoethanol [Millipore], 1000 U/mL LIF supplement [ESGRO, Millipore]). Experiments comparing wild-type versus *Dux*^*−/−*^ E14 ESCs were performed in FBS/LIF media as above, supplemented with 2i (1 µM PD0325901 [Stem Cell Technologies], 3 µM CHIR99021 [Cambridge Bioscience]) as in Grow et al. (2021). ESCs were routinely tested for mycoplasma and found to be negative. Inhibitors (Supplemental Table S2) were added to ESCs at the indicated concentrations and durations unless otherwise explicitly mentioned in the figure legends.

### 2C-GFP/CD4 cell line

The 2C-GFP reporter construct (Ishiuchi et al. 2015) was modified to insert a T2A cleavage element followed by the extracellular portion of mouse CD4 (amino acids 1–427) immediately downstream from GFP so that activation of MERVL in the 2C-like state labeled cells doubly positive for GFP and CD4. ESCs were negative for CD4 expression, enabling rapid purification of endogenous 2C-like cells via selection for CD4^+^ surface expression. E14 ESCs were nucleofected with 4 µg of linearized 2C-GFP plasmid, plated at low density in 10-cm^2^ plates, and then selected with 250 µg/mL G418 (Mirus) for 8 d. Individual colonies were picked and expanded, with a single colony that showed high specific expression of GFP expanded and used for subsequent validations and experiments. For 2C-GFP/CD4 isolation, cells were trypsinized, washed, resuspended in FACS buffer (PBS, 3% FBS, 1 mM EDTA), and then isolated either by MACS using CD4 (L3T4) microbeads (Miltenyi Biotec) or with the EasySep mouse CD4-positive selection kit II (Stem Cell Technologies) according to the manufacturer's protocols in each case. Apart from Supplemental Figure S1, all purification experiments were performed with the EasySep kit. Flow-through cells were collected as the 2C-negative population. For flow cytometry analysis, ESCs were pelleted and resuspended in FACS buffer containing 1:8000 Sytox Blue (Thermo Fisher Scientific) to enable exclusion of dead cells.

### siRNA-mediated knockdown

The nucleolar miniscreen was performed with a Cherry Pick custom library plate of OnTargetPlus siRNAs (Horizon Discovery) consisting of 20 wells of different gene targeting siRNA pools and three siControl wells. 2C-GFP ESCs (Percharde et al. 2018) at a density of 10,000 cells per 96-well plate were transfected in suspension with 3 pmol of siRNA and 0.17 µL of Lipofectamine 2000 per well of a 96-well plate and incubated overnight. The medium was changed the following day, and then cells were cultured for a further 2 d before analysis. For flow cytometry, ESCs were trypsinized, transferred to a 96-well round-bottom plate, pelleted, washed, and resuspended in PBS plus 1:8000 Sytox Blue (Thermo Fisher Scientific), and then the percentage of GFP in live cells was analyzed by flow cytometry on a BD Fortessa cytometer. The nucleolar miniscreen was performed in triplicate wells, and the entire experiment was repeated on a different day with highly similar results. *Z*-scores were calculated as the percentage 2C-GFP value for each factor minus the average 2C-GFP level for the entire plate, divided by the plate standard deviation. Other siRNA transfections or validation experiments were performed as above, scaling up cell numbers, Lipofectamine, and siRNA amounts accordingly for ESCs cultured in 12- or 24-well plates, with cells harvested at the indicated time points.

### Nascent transcription/translation assays

Nascent transcription (EU) and translation (HPG) assays were carried out as described previously (Percharde et al. 2018) using Click-iT assay kits (Thermo Fisher Scientific) according to the manufacturer's protocol. For HPG assays, ESCs were cultured in medium made with DMEM lacking methionine (Gibco 21013024) for 1 h prior to HPG addition. ESCs were cultured with 1 mM EU or 50 µM HPG for 45 min before fixation, permeabilization, and Click-iT reaction. Where indicated, immunofluorescence labeling was carried out prior to Click-iT as described above with the exception that primary antibodies were added for 1–2 h at room temperature.

### Western blotting

Whole-cell extracts were prepared from ESCs by scraping in ice-cold RIPA buffer containing protease inhibitors (Halt), incubating for 30 min at 4°C, and then pelleting at 16,000*g* for 20 min to remove insoluble material. Proteins were quantified by the BCA assay (Pierce), and equal amounts were loaded onto 4%–12% Bolt Bis-Tris plus SDS-PAGE gels (Thermo Fisher) to separate proteins and then transferred onto PVDF membranes. Blocking was performed in 5% milk/PBS-T for 1 h, and then membranes were incubated overnight with primary antibodies at 4°C in milk/PBS-T. The next day, membranes were incubated with the appropriate HRP-conjugated secondary antibodies (Cell Signaling) for 1 h, and then proteins were detected by ECL reagent on an Amersham Imager 680.

### RNA extraction and expression analysis

RNA was isolated directly from ESCs by scraping in RLT lysis buffer (Qiagen) containing 1:100 β-mercaptoethanol (Sigma), or RLT was added to equal numbers of ESCs for CNN approaches. RNA was purified and DNase I-treated according to the manufacturer's instructions using RNeasy mini kits (Qiagen). For embryo inhibitor experiments, two-cell embryos were flushed at 46 h after hCG and cultured in KSOMaa medium containing either 1 µM CX-5461, 1 µM BMH-21, or 0.1% DMSO in a four-well dish. Culture in inhibitors began after 1 h for a period of 8 or 24 h. Equal numbers of embryos per experimental condition were lysed in 75 µL of buffer RLT prepared as above, and the RNA was isolated according to RNeasy micro kits (Qiagen). In ESCs and embryos, cDNA synthesis was performed with up to 1 µg of DNase-treated RNA using a high-capacity RNA-to-cDNA kit (Thermo Fisher Scientific), and qRT-PCR was performed with SYBR Green (KAPA) on a QuantStudio 5 qPCR machine (Thermo Fisher Scientific). qRT-PCR data were normalized to two housekeeping genes (Rpl7/H2A), unless a cell number normalization (CNN) approach was used as detailed in the legend for Figure 4, A, G, and H. Primer sequences are described in Supplemental Table S2.

### RNA sequencing and analysis

For RNA-seq, RNA was extracted using the RNeasy mini kit as for qRT-PCR, and then three biological replicates per condition of DNase-treated total RNA spiked with ERCCs (Thermo Fisher) were used for RNA-seq library preparation and sequencing at the MRC London Institute of Medical Sciences Genomics Core facility. RNA quality was assessed using the Agilent 2100 RNA 6000 Nano assay, and libraries were prepared using the NEBNext Ultra II directional RNA library preparation kit with NEBNext poly(A) mRNA magnetic isolation module following the manufacturer's instructions. Library quality was evaluated using the Agilent 2100 high-sensitivity DNA assay, and their concentrations were measured using the Qubit dsDNA HS assay kit. Libraries were pooled in equimolar quantities and sequenced on an Illumina NextSeq 2000 to generate a minimum of 40 million single-read 50-bp reads (with unique 8-bp dual indexes) per sample. Reads were trimmed and aligned to reference genome mm10 plus ERCCs using TopHat2. Default settings were used apart from the specification “g -1” to map each multimapping read to one random TE or gene in the genome. Reads were counted using the Subread package FeatureCounts for each gene or TE family. Data were filtered to exclude rows with counts per million (cpm) >0 in fewer than three samples. To account for any global decreases in RNA amounts due to iPol I, we used our previously described cell number-normalized (CNN) approach to normalize reads to the abundance of ERCC spike-ins (Percharde et al. 2017) using Limma Voom. All other RNA-seq analyses and statistics were performed in R/Bioconductor. Normalized RNA-seq expression data are in Supplemental Table S1. RNA-seq data have been uploaded to GEO, accession GSE185424.

### ESC immunofluorescence

ESCs were allowed to adhere to Matrigel-coated eight-well chambers or 10-mm glass coverslips for 1–2 h, fixed in 4% PFA for 10 min, and then stored in PBS until staining. Blocking and permeabilization were carried out in one step in immunofluorescence (IF) buffer (PBS, 10% donkey serum, 2.5% BSA) plus 0.4% Triton X-100 for 30 min. Primary antibody incubations were carried out overnight at 4°C using the indicated antibodies and dilutions in IF buffer as described in Supplemental Table S2. The next day, samples were washed with PBS and incubated in secondary antibodies (1:500 Alexa 488 nm-, 594 nm-, or 647 nm-conjugated antibodies) for 1 h at room temperature, followed by a wash for 30 min in PBS plus DAPI and two more washes in PBS, and then samples were mounted in VectaShield mounting medium containing DAPI. Confocal images were taken on a Leica SP5 fluorescent microscope under an oil immersion 63× objective.

### Embryo in vitro culture EU/HPG and IF experiments

Embryos were fixed in 4% PFA in PBS containing 0.1% Triton X-100 for 30 min, followed by three washes in PBS containing 0.1% PVA (PBS-PVA). Samples were permeabilized in PBS containing 0.5% Triton X-100 for 30 min, followed by blocking in 5% BSA in PBS for 1.5 h. A 1:100 dilution of primary antibody (rabbit antinucleolin; Abcam ab22558) was prepared in blocking solution, and embryos were incubated in 10-µL drops in a humidified Terasaki plate (Greiner Bio-One) overnight at 4°C. Embryos were washed three times in PBS containing 0.1% Tween-20 and 0.1% PVA (PBST-PVA) for 5 min. A 1:500 dilution of secondary antibody (donkey antirabbit Alexa Fluor 488; Thermo Fisher A21206) was prepared in blocking solution, and embryos were incubated in 10-µL drops in a humidified Terasaki plate for 1 h in the dark. EU and HPG assays were performed as for ESCs, except that embryos were incubated for 1 h in pre-equilibrated KSOM (without amino acids; Millipore) prior to incubation in KSOM containing 500 µM HPG or 1 mM EU for 2 h. Embryos were fixed in 4% PFA in PBS containing 0.1% Triton X-100 for 15 min and permeabilized in 0.5% Triton X-100 in PBS for 20 min prior to Click-iT reactions. Prior to mounting, all samples were washed three times in PBST-PVA for 5 min, followed by a 30-min incubation in 1:1000 DAPI in PBS and a further three washes in PBS-PVA. Embryos were mounted in VectaShield (Vector Laboratories) under a 20-mm × 20-mm #1.5 coverslip (Agar Scientific) supported at the corners by Dow Corning high-vacuum silicone grease (Sigma-Aldrich) and sealed with nail polish. Confocal images were captured using a Leica SP5 or SP8 fluorescence microscope using an oil immersion 40× objective and acquired in 1-µm Z-stacks. All steps were carried out in 500-µL volumes at room temperature unless otherwise noted.

### DNA FISH with immunofluorescence (immuno-DNA FISH)

Immunofluorescence detection of nucleolus and nuclear lamina combined with DNA was performed essentially as described previously (Beagrie et al. 2017). Briefly, ESCs were fixed with 4% plus 0.1% Triton in PBS for 10 min, washed three times in PBS, equilibrated in 20% glycerol in PBS three times for 10 min, then subsequently frozen in liquid nitrogen, and stored at −80°C. After thawing, cells were washed in PBS three times, permeabilized for 10 min with 0.1% Triton in PBS, and blocked with 2% BSA-PBS for 30 min before immunolabeling. Cells were incubated with the indicated mouse anti-B23 as above, and rabbit anti-laminB1 (1:2000; Abcam ab16048) antibodies in 2% BSA in PBS for 2 h, and then detected with Alexa Fluor 488- or 647-conjugated antibodies. After immunolabeling, cells were fixed with 4% PFA in PBS for 30 min prior to FISH to preserve immunocomplexes during FISH. *Dux* oligo probes used to detect *Dux* foci were used and labeled with Cy3 fluorphores (Amersham PA23001) as described previously (Percharde et al. 2018). For hybridization, 1 µg of mouse Cot1 DNA (Invitrogen 18440), 10 µg of salmon sperm DNA (Invitrogen 15632011), and 3 μL of Cy3- labeled Dux oligos were precipitated and resuspended in 6 μL of hybridization buffer (Sigma-Aldrich H7782) ready for DNA FISH. Immunolabled cells were rinsed three times in PBS, incubated for 15 min in 20 mM glycine in PBS, rinsed three times in PBS, permeabilized for 10 min with 0.2% Triton, and then washed again. Cells were incubated for 1–2 h at 37°C with 250 µg/mL RNase A (Sigma) in 2× SSC, treated for 10 min with 0.1 M HCl, dehydrated in ethanol 50%–100% series for 3 min each, dried briefly, denatured for 10 min at 80°C in 70% deionized formamide in 2× SSC, and then redehydrated as above. After a brief period of drying, coverslips were overlaid onto probe mixture on Hybrislips (Molecular Probe by Life Technology H18200) and sealed with Fixogum rubber cement (MP Biomedicals 11FIXO0125) for in situ hybridization. Hybridization was carried out in a moist chamber for at least 40 h at 37°C. Posthybridization washes were as follows: 40% formamide in 2× SSC three times for 10 min, 2× SSC three times for 10 min, and 3× PBS. Nuclei were counterstained with 1 µg/mL DAPI in PBS for 30 min and mounted in VectaShield (Vector Laboratories H-1000) immediately prior to imaging.

### Superresolution structured illumination microscopy (SIM)

Purified 2C-GFP/CD4-negative and -positive cells or wild-type ESCs incubated for 8 h with or without iPol I were adhered onto Matrigel-coated µ-slide eight-well glass-bottom dishes (Ibidi 80827), fixed with 4% paraformaldehyde in PBS for 10 min, and then stored in PBS at 4°C before immunolabeling. Cells were rinsed three times in PBS, incubated for 15 min in 20 mM glycine in PBS, rinsed, permeabilized with 0.1% Triton X-100 for 10 min in PBS, blocked for 1 h with 4% BSA in PBS, and then incubated with mouse anti-B23 to detect nucleoli in 4% BSA in PBS overnight at 4°C. The next day, samples were washed three times for 60 min with 2% BSA in PBS, then incubated for 2 h with Alexa secondary antibodies (1:250), and washed again as above. Finally, samples were washed and counterstained with 1 µg/mL DAPI for 30 min and rinsed successively in PBS before coverslips were mounted in VectaShield. The long incubation times used allowed for antibody accessibility throughout the cells, providing the highest sensitivity for SIM imaging. Multicolor SIM imaging was performed using a Zeiss Elyra S.1 (Carl Zeiss Microimaging) and a plan-apochromat 63×/1.4 oil lens. Raw SIM images were acquired with an sCMOS camera (pco.Edge 4.2) using five phase shifts and three grid rotations, with a *z* step size of 0.1 μm. Different fluorescent labels were acquired sequentially using 642-, 561-, 488-, and 405-nm laser lines. SIM images were reconstructed with ZEN 2012 SP4 (black) software (Carl Zeiss Microimaging, version 13.0.2.518) using default parameter settings. Channel alignment was performed using calibrations obtained from a multicolored bead slide, acquired with equivalent acquisition settings.

### ESC confocal microscopy and quantitative image analysis

Multicolor image single snapshots (IF and EU/HPG assays) or Z-stacks (250-nm step size, RNA/DNA FISH) were acquired with a laser-scanning confocal microscope with a pinhole diameter of 1 airy unit (Leica TCS SP5 or SP8; objective lens: 63×/1.40 NA oil CS2 HC PL APO; laser lines: 405/488/552/638 nm). Different channels were imaged sequentially to avoid bleedthrough and cross-excitation and then exported as TIFF files for further image analysis. RNA and DNA FISH image Z-stacks were also acquired on an Olympus spinning-disk confocal system based on an IX83 inverted microscope stand (Yokogawa CSU-W1 scan head with 50-µm diameter pinhole disk; objective lens: 60×/1.40 NA Plan-Apo; Hamamatsu ORCA Flash 4.0 V2 camera, step size 200 nm). Raw .LIF images were processed into TIFF files and merged, each channel manually thresholding or filtering with the same setting in Fiji software for data analysis.

Dux FISH 3D spatial analysis was undertaken using custom-written scripts in Fiji for nucleus and nucleolus segmentation, and Imaris (version 9.6.0, Bitplane AG) for Dux FISH probe identification and distance measurements. Briefly, identification of LaminB1-labeled nuclei and B23-labeled nucleoli was performed in Fiji in combination with the MorphoLibJ plugin to perform 3D segmentation. Labeled image masks generated were combined with the original image stack and imported into Imaris for use with the “Cells” package to facilitate interactive review of 3D segmentation results and Dux FISH probe identification. Identified *Dux* FISH loci were related to each individual nucleus and contained nucleoli, and distance to the nearest nucleolus and nucleus border were measured.

The analysis of nucleolar morphology and fluorescence intensities was carried out with a custom-written CellProfiler pipeline (https://cellprofiler.org; software version 4.1.3). To describe and quantify the number of “ring-shaped” nucleoli (showing a distinct fluorescent B23 signal at the rims of the nucleoli with a much dimmer interior signal), the fluorescence intensity distribution over four concentric layers in each nucleolus was measured. The mean fluorescence intensity of the outer layer was divided by the mean intensity of the innermost layer, and any nucleolus with a ratio >1.4 was counted in the “ring-shaped” category. Nucleolar circularity (“FormFactor”) was calculated within CellProfiler according to 4π × (area)/(perimeter)^2^, excluding very small nucleoli, which were liable to give unreliable measurements (FormFactor values > 1).

### Statistical analysis

All statistical analyses were carried out in Prism 7 or above (Graphpad) or R (RNA-seq data). Details of individual tests are outlined in each figure legend, including number and type of replication performed (*n*) and the reported error either as standard deviation (SD) or standard error of the mean (SEM). All statistics are *P* < 0.05 (*), *P* < 0.01 (**), *P* < 0.001 (***), and *P* < 0.0001 (****), with the relevant test performed described in the figure legends and corrections for multiple testing applied where necessary. Welch's correction was applied to *t*-tests when the variance was unequal between conditions.

### Data availability

RNA-seq data have been uploaded to GEO, accession GSE185424.

### Code availability

RNA-seq data were analyzed with standard packages and programs, as detailed in the Materials and Methods. Code for data processing and analysis are available at https://github.com/mpercharde/RNAseq and/or are available on request.

## Supplementary Material

Supplemental Material
