# Supplementary material for: Nucleolar-based Dux repression is essential for embryonic two-cell stage exit
Source: Genes Dev. 2022 Mar 1;36(5-6):331–47. doi: 10.1101/gad.349172.121 (PMC8973846; doi:10.1101/gad.349172.121)
Supplement: Supplemental Material [file supp_36_5-6_331__DC1.html]

Nucleolar-based Dux repression is essential for embryonic two-cell stage exit — Supplemental Material 

# Nucleolar-based *Dux* repression is essential for embryonic two-cell stage exit

## Supplemental Material

- Supplemental\_Figures.pdf
- SupplementalTableS1.xlsx
- SupplementalTableS2.xlsx
