# Supplementary material for: Nucleolar-based Dux repression is essential for embryonic two-cell stage exit
Source: Genes Dev. 2022 Mar 1;36(5-6):331–47. doi: 10.1101/gad.349172.121 (PMC8973846; doi:10.1101/gad.349172.121)
Supplement: Supplemental Material [file supp_gad.349172.121_Supplemental_Figures_.pdf]

Nucleolar-based Dux repression is essential for 2-cell stage exit

Supplemental Information:

Supplemental Figures 1-8
Supplemental Methods

Table S1: RNA-seq data in this study upon iPol I
Table S2: Reagents, probes and primer sequences used in this study

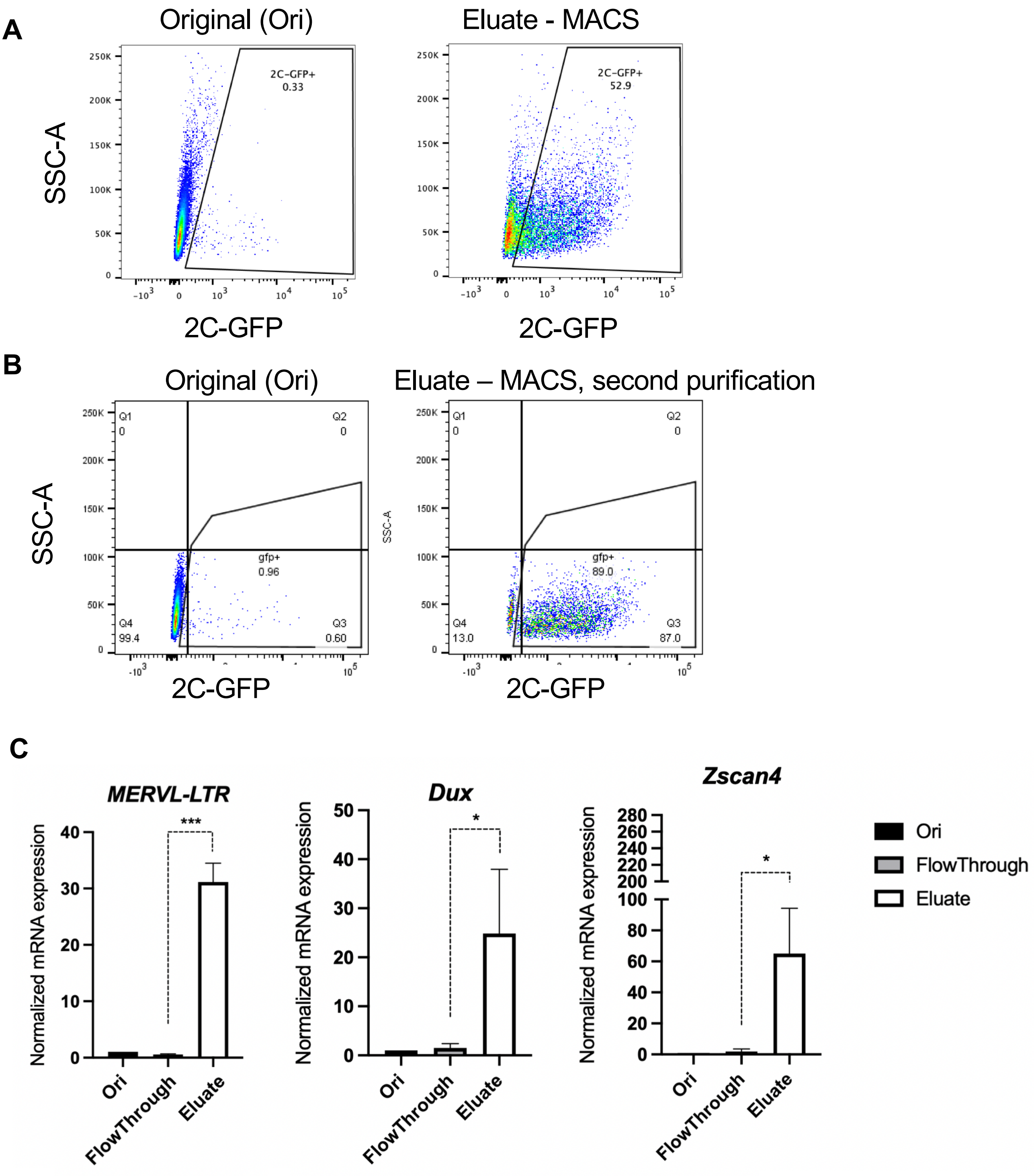

Supplemental Fig. S1. MACS purification of 2C-like cells.

A) Representative flow cytometry plots of 2C-GFP/CD4 ESCs before and after MACS purification
B) Flow cytometry plots showing increased purity of 2C-GFP+ cells after two rounds of MACS purification.
C) qRT-PCR analysis of 2C-specific transcripts after MACS purification. Note that 2C gene induction with MACS is lower than CD4 column-free, bead-based purification (StemCell Inc) (Figure 1D). CD4 column-free isolation was therefore used in all subsequent experiments throughout this study. Data are mean +/- s.e.m, 3 independent experiments, P values, unpaired Student's t-test.

Figure S2 **Xie et al**

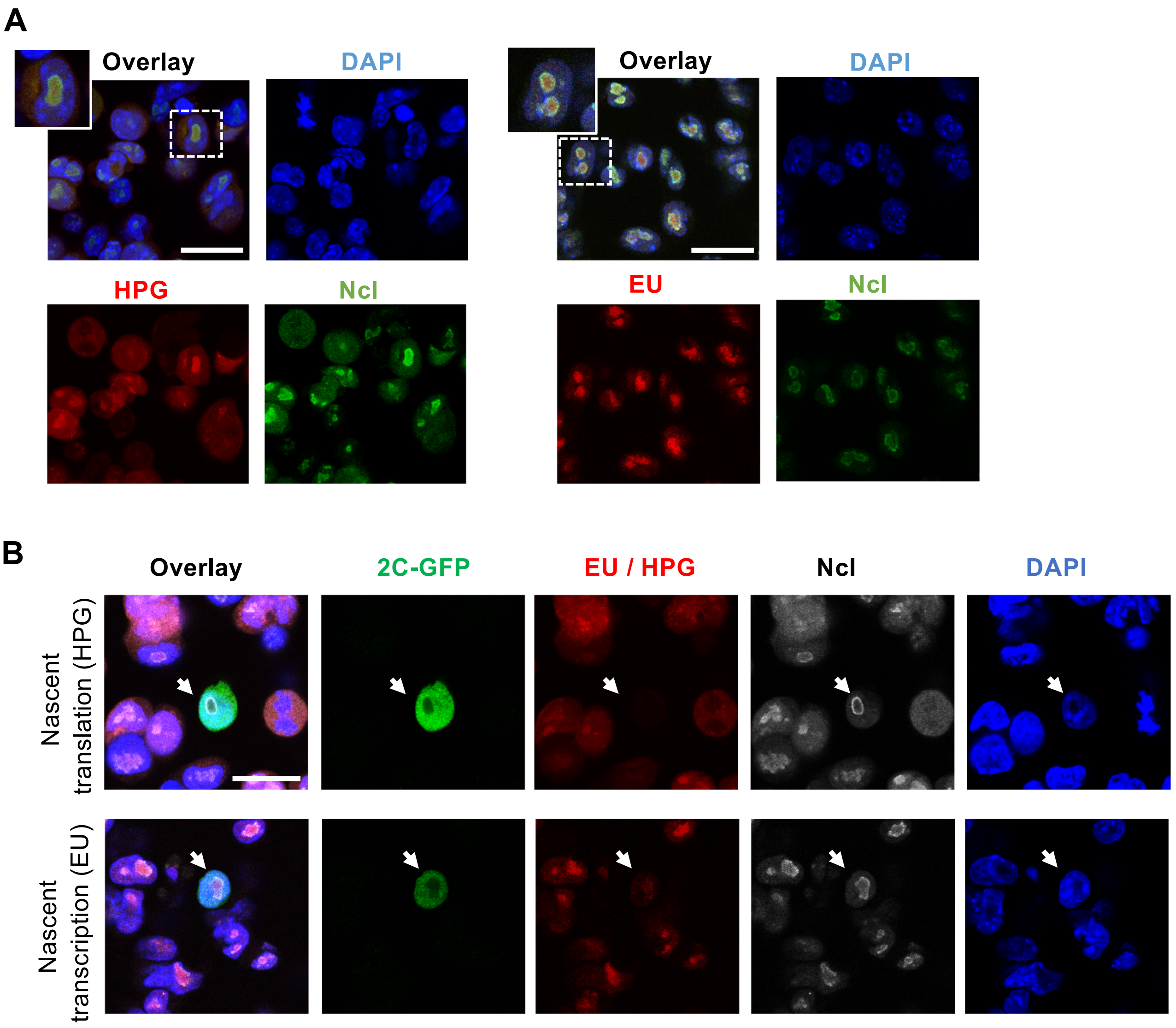

Supplemental Fig. S2. Nascent nucleolar RNA and protein expression.

A) Representative confocal microscopy images in WT ESCs confirming overlap of Nucleolin (Ncl) and HPG (left) or EU (right) in ESCs. Scale, 25 μm.

B) Representative confocal microscopy images of EU/HPG incorporation in A1 2C-GFP reporter cells. White arrows denote 2C-like cells. Scale, 25 μm.

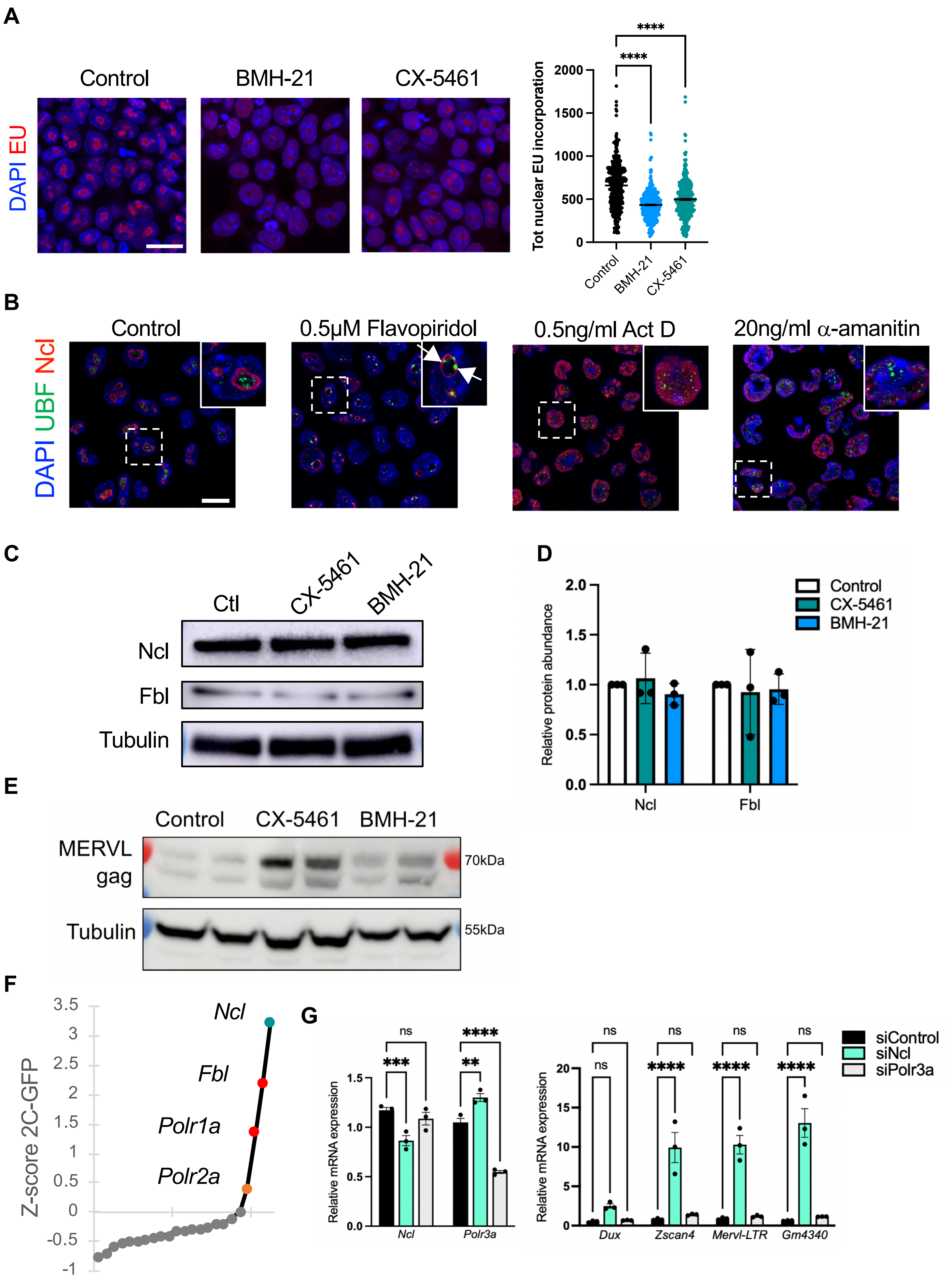
A
Control
BMH-21
CX-5461
DAPI EU
Tot nuclear EU incorporation
****
****
2000
1500
1000
500
0
Control
BMH-21
CX-5461
B
Control
0.5μM Flavopiridol
0.5ng/ml Act D
20ng/ml α-amanitin
DAPI UBF Ncl
C
Ctl
CX-5461
BMH-21
Ncl
Fbl
Tubulin
D
Relative protein abundance
2.0
1.5
1.0
0.5
0.0
Control
CX-5461
BMH-21
Ncl
Fbl
E
Control
CX-5461
BMH-21
MERVL gag
Tubulin
70kDa
55kDa
F
Z-score 2C-GFP
3.5
3
2.5
2
1.5
1
0.5
0
-0.5
-1
Ncl
Fbl
Polr1a
Polr2a
G
Relative mRNA expression
1.5
1.0
0.5
0.0
ns
***
****
**
Ncl
Polr3a
Relative mRNA expression
20
15
10
5
0
ns
****
Dux
Zscan4
Mervl-LTR
Gm4340
siControl
siNcl
siPolr3a

Supplemental Fig. S3. Nucleolar disruption by RNA Pol I inhibition.

A) Representative confocal microscopy images and quantification of nascent RNA levels (EU incorporation) following 2h RNA Pol I inhibition. Data are mean +/- s.e.m, P values, one-way ANOVA with Dunnett multiple comparisons correction. Scale bars, 25 μm.
B) Representative confocal microscopy images of ESC nucleolar proteins and morphology 8h following the indicated Pol I/II inhibitors. Nucleolar caps are indicated with arrowheads. Scale bars, 20 μm.
C) Western blots of Ncl/Fbl protein after 8h iPol I treatment, representative of 3 experiments.
D) Quantification of relative amounts of Ncl/Fbl protein relative to Gapdh/Tubulin loading controls, data are mean +/- s.e.m from 3 experiments.
E) Western blot of MERVL gag protein induction upon overnight iPol I treatment.
F) Z-scores for 2C-GFP cell induction following nucleolar protein knockdown, at d3 following transfection.
G) qPCR expression data for the indicated genes 72h after siRNA-mediated knockdowns. Data are mean +/- s.e.m, n =3 biological replicates, representative of 2 independent experiments. P values, 2-way ANOVA with Dunnett's multiple comparisons test.

Figure S4

Xie et al

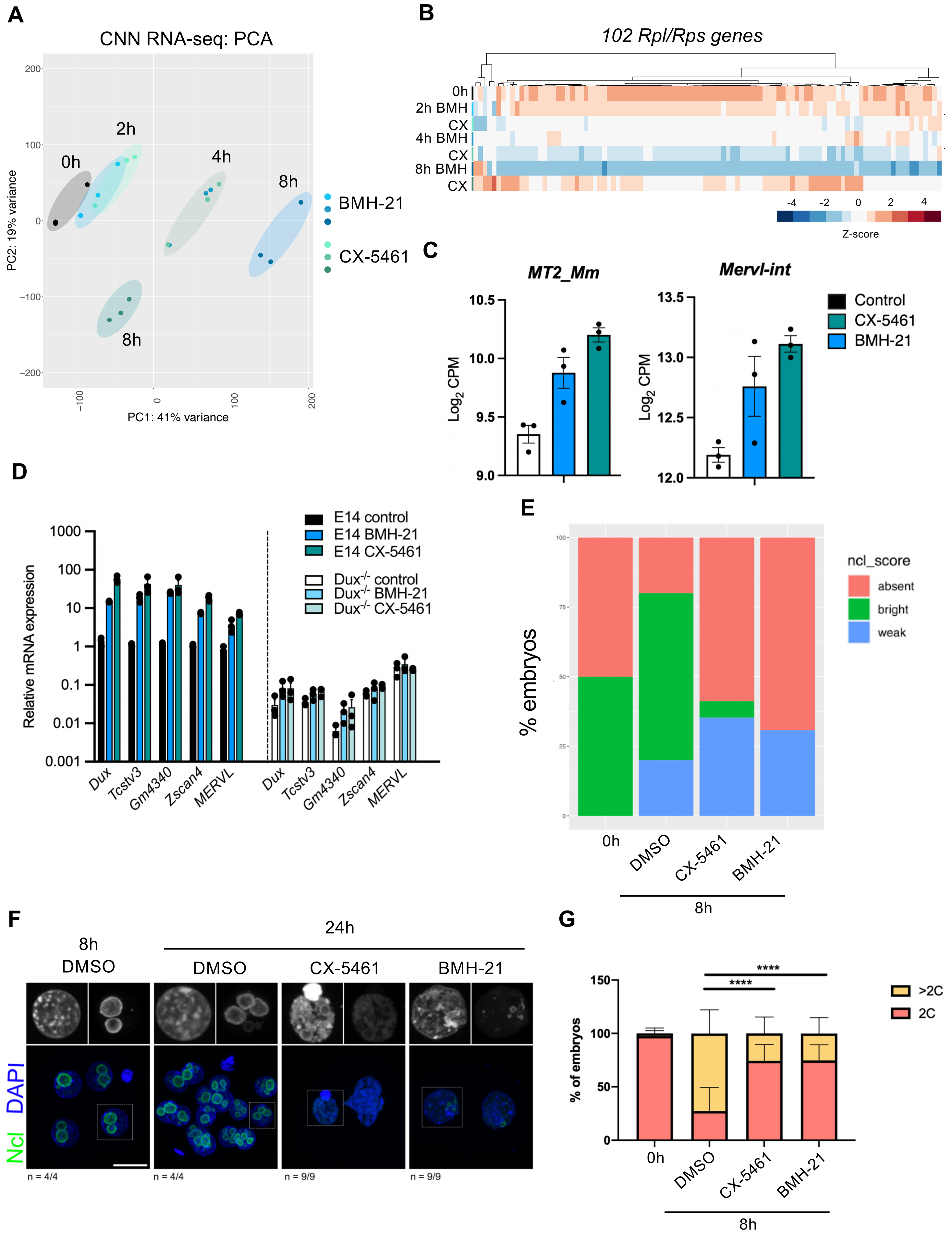

Supplemental Fig. S4. *Dux* activation upon iPol I in ESCs and embryos

A) PCA of the first 2 principle components of CNN RNA-seq data following a timecourse of Pol I.

B) Heatmap of Rpl/Rps genes showing a global decrease following various durations of iPol I. We noted in (A-B) there is some evidence of partial reduction in inhibition by CX-5461 (CX) by 8h; this may explain a lower induction of *Dux* compared to BMH-21 (BMH) at this timepoint (Figure 4A) and could be due to reduced stability or feedback mechanisms. Conversely, 2C-specific gene upregulation is lower at 16-24h BMH-21 (Figure 3D), which may be due to apoptosis caused by abnormally high *Dux* expression.

C) Normalised log2 RNA-seq expression data showing upregulation of TEs belonging to the MERVL family at 8h following iPol I. CPM, counts per million.

D) Relative expression of *Dux* and 2C-like genes in wt E14 ESCs compared to E14 Dux-/- ESCs (Grow et al., 2021) after 16-24h iPol I. All data are shown relative to E14 wt control and are mean +/- s.e.m, n=3 biological replicates, representative of 2 experiments.

E) Proportion of embryos showing absent, faint, or bright Nucleolin (Ncl) staining before and after 8h iPol I, from 2 experiments.

F) Ncl immunofluorescence in mid-2C embryos fixed immediately or cultured for 24h with the indicated inhibitors. N=number of embryos with the representative staining, from one experiment, scale, 20μm.

G) Developmental progression rates past 2-cell before and 8h after the indicated treatments. P values, Fisher's exact test, >50 embryos from 4 experiments per condition.

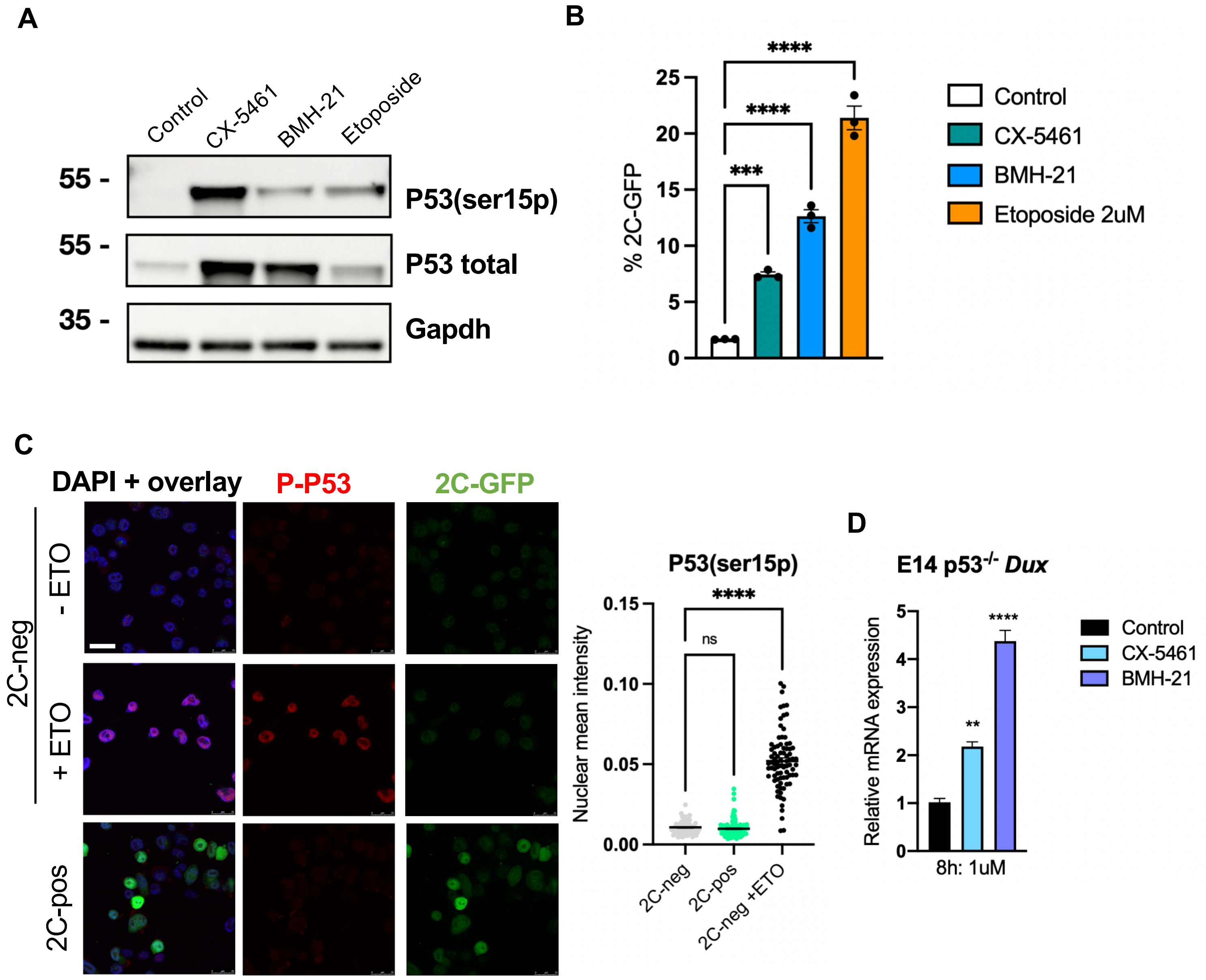

Supplemental Fig. S5. Mechanism of Dux and 2C activation upon iPol I

A) Western blots for total and phospho-p53 (Ser15p) in ESCs 24h after the indicated inhibitor treatments. Data are representative of 2 experiments.
B) % 2C-GFP+ cells as quantified by flow cytometry, 24h after treatment with the indicated inhibitors. Data are mean +/- s.e.m, n= 3 biological replicates.
C) Representative confocal microscopy images and quantification of phospho-p53 levels in 2C-negative and 2C-positive cells. Etoposide treatment was for 4h in 2C-negative cells as a positive control for p53 activation. Scale bars, 25 μm.
D) qRT-PCR analysis of *Dux* upregulation after 8h iPol I in p53$^{-/-}$ ESCs, data are mean +/- s.e.m, 3 biological replicates.

All P values, one-way ANOVA with Dunnett multiple comparisons correction.

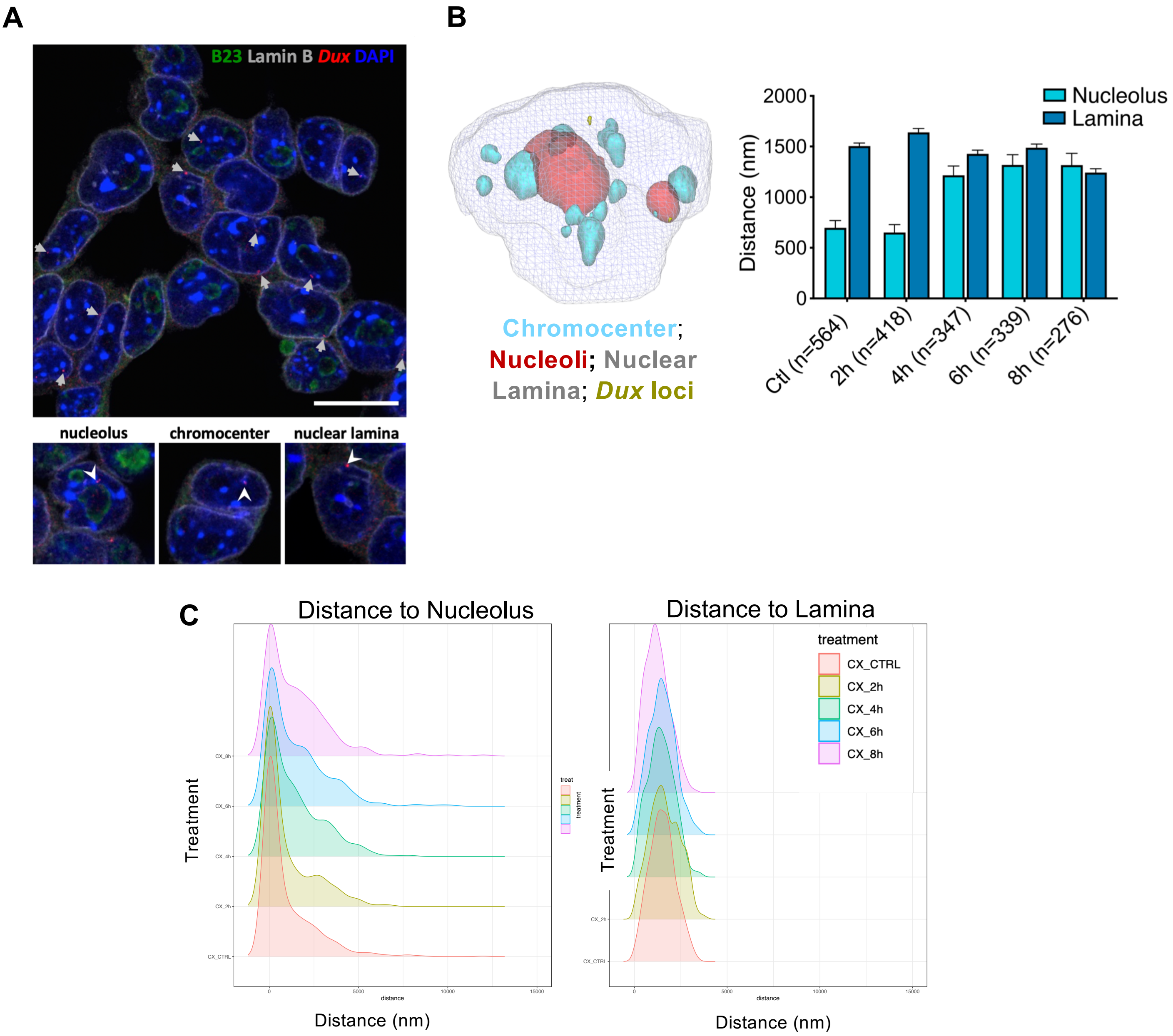

Supplemental Fig. S6. *Dux* movement upon iPol I.

A) Representative immuno-DNA-FISH for *Dux* DNA in ESCs alongside nucleolar and lamina markers with localization classification (below). *Dux* loci are marked by white arrowheads. Scale, 20µm

B) Example 3D reconstruction and identification of nuclear compartments and *Dux* loci in Imaris for calculation of 3D distances, and quantification of *Dux* movement from the nucleolus or lamina upon CX-5461 treatment. Data are mean +/- s.e.m for n= number of cells indicated.

C) Density plots of *Dux* distance (in nm) to the lamina or nucleolus upon CX-5461.

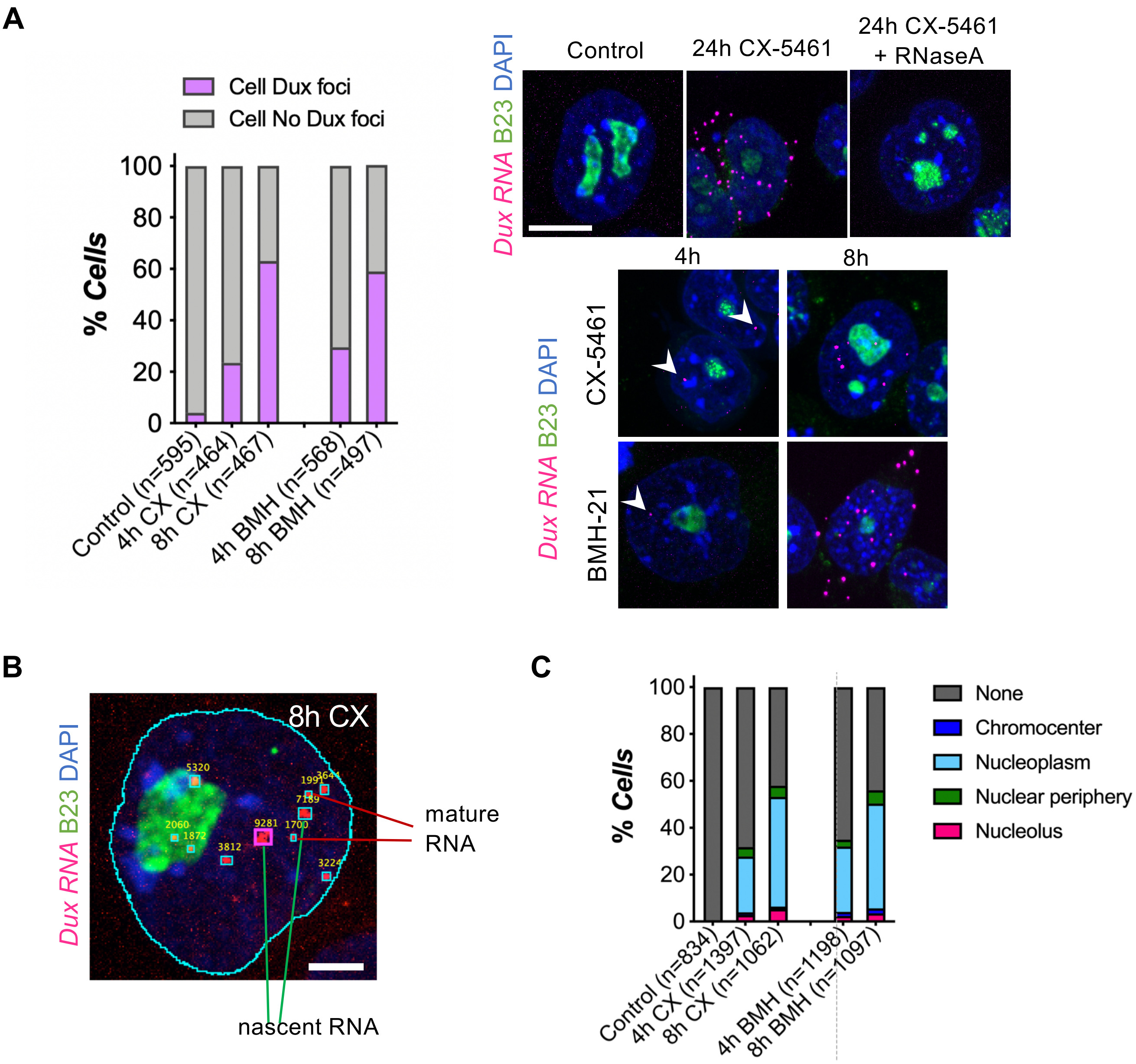

Supplemental Fig. S7. Single-cell analysis of *Dux* expression upon iPol I.

A) RNA FISH analysis of *Dux* expression in individual cells upon CX-5461 (CX) or BMH-21 (BMH) treatment. N = number of cells scored. White arrowheads indicate putative nascent RNA foci. Scale bars, 10 μm.

B) Example identification of putative nascent RNA (up to 2 largest foci, Mueller et al., 2013) associated with the transcriptional start site, in cells with >2 RNA foci. Numbers correspond to integrated intensity measurements. Scale, 4μm

C) Location of putative nascent RNA foci in ESCs upon iPol I, n = number of cells scored.

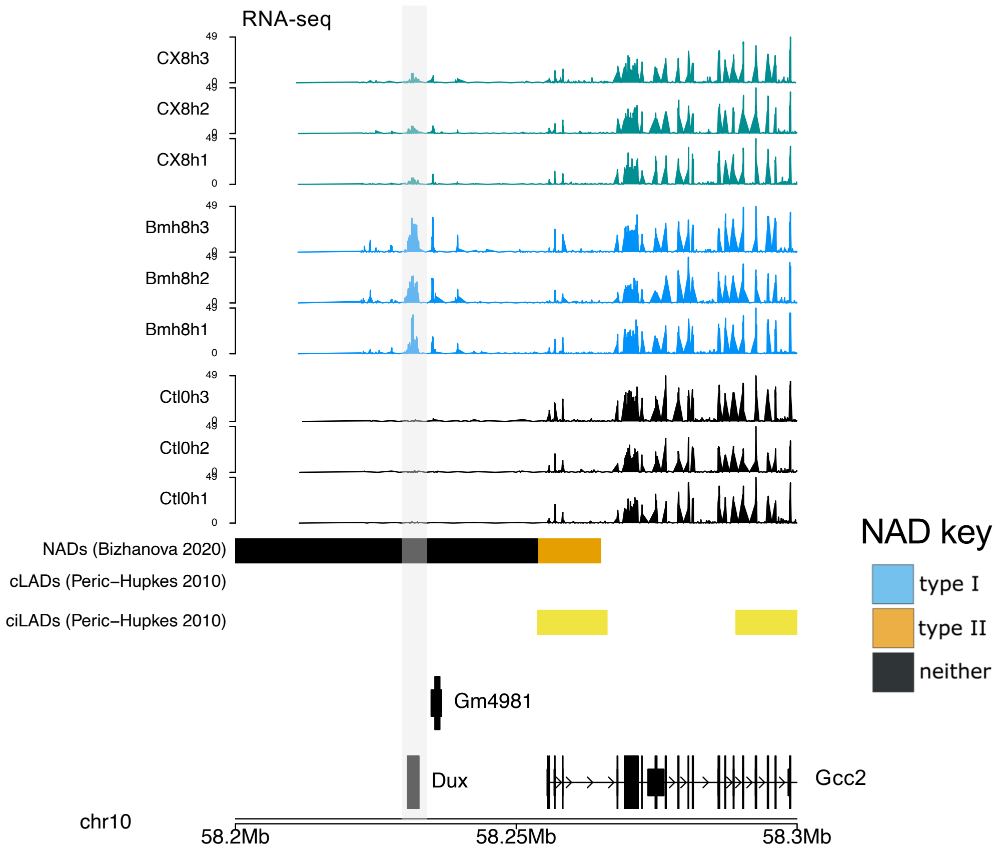

Supplemental Fig. S8. Dux NAD localisation

Browser screenshot of the indicated region of chr10 containing the *Dux* locus (highlighted) and neighbouring genes with overlayed RNA-seq tracks, confirming that *Dux* is situated in a NAD as defined in (Bizhanova et al., 2020). *Dux* is considered within a “neither” NAD, because its NAD does not overlap a cLAD or ciLAD - meaning in some cell types it is also lamina associated. This is consistent with some *Dux* loci positioning at the lamina in ESCs (Fig 5).

Supplemental Materials and Methods

Single Molecule RNA FISH combined with immunofluorescence staining (Immuno-smRNA-FISH)

Dux smRNA-FISH was based on branched DNA technology (https://www.thermofisher.com/uk/en/home/life-science/cell-analysis/cellular-imaging/in-situ-hybridization-ish/rna-fish.html) to detect the dynamics of Dux expression upon the inhibition of ribosomal RNA synthesis. A target set of 20 short Alexa647 labelled oligo mouse Dux ViewRNA ISH probe (VB6-3223670-VC, type-6; ThermoFisher Scientific) was designed and used to hybridize to Dux RNA sequence. Cells were first adhered on Matrigel-coated 10 mm glass coverslips and fixed with 4% paraformaldehyde in DEPC-treated PBS, containing 2.5% Acetic acid for 10 mins. Immunolabeling with performed with mouse anti-B23 primary antibody (1:300; 2h) then detected with Alexa 488 donkey anti-mouse antibody (Invitrogen; 1:500; 1h), then further fixed with 4% paraformaldehyde in DEPC-PBS, 10 min to preserve immunocomplexes before FISH. smRNA FISH was carried out in accordance with the manufacturer's protocol for the ViewRNA TM ISH Cell Assay (QVC001; ThermoFisher Scientific). Briefly, previously immunolabled cells were washed in PBS (3x), permeabilised (1:2 digestion solution in DEPC-PBS; 5 min), protease treated (1:8000 in DEPC-PBS, 10 min), and subsequently hybridized with Alexa 647 ViewRNA ISH Dux probe (P4, 1:100 in QF diluent; 3h, 40oC). Following probe hybridisation, cells were washed and then subjected to sequential hybridisation with pre-amplifier DNA, amplifier DNA and fluorophore labeled in provided diluent (1:25, 1h, 40oC each step). After detection, cells were washed and nuclei were stained with 1μg/ml DAPI in PBS before imaging. Control experiments were performed with either hybridisation with mouse B-actin probe as a positive control that revealed abundant b-actin RNA foci throughout nucleus and cytoplasm, or Rnase A treatment (250 μg/ml in PBS, 2h, 37oC) prior to hybridisation, which abrograted Dux signals.

Nascent RNA foci quantification

For identification of nascent RNA, integrated intensity measurements were initially taken for all identified RNA probes, in Imaris. After calculation of the population mean integrated intensity, multiple intensity thresholds were calculated in increments of population standard deviation above the mean. RNA probe intensities that fell within calculated thresholds were visually highlighted in the raw image data for further validation and classification. For scoring of the location of putative nascent foci, these were identified as the ≤ 2 brightest/largest spots per nucleus in cells with multiple foci. In cells with ≤ 2 foci present per cell, for example after 4h iPol I (where very few cells have >2 foci), these loci were treated as "putative nascent".

Embryo DNA-FISH

The genomic region of Dux locus was detected using fosmid probes (WI1-1484C16; Genomic Coordinates: chr10:57682337-57718286) from BACPAC Resources Center (https://bacpacresources.org/; California, USA). Probes were labelled with tetramethyl-rhodamine-5-dUTP (Roche) by nick translation (Roche), and purified from unincorporated nucleotides using MicroBioSpin P-30 chromatography columns (Bio-Rad). Different stages of embryos were attached on poly-l lysine coated coverslips first, fixed with 4%PFA + 0.1% Triton in PBS (30 min), permeabilized with 0.5% triton in PBS (30min), and then processed for FISH as for ESCs. Embryo Immuno-DNA-FISH was performed directly on fresh fixed embryos or on embryos previously immunolabelled with antibody against B23 to mark the nucleolus as above. Hybridization was carried out as described, and hybridizaiton mixtures contained 1 μg mouse Cot1 DNA, 10 μg salmon sperm DNA and the appropriate 4 ul tetramethyl-rhodamine-5-dUTP nick-translated Dux fosmid probe, respectively. Before hybridization, probes were precipitated and resuspended in 6 μl of hybridisation buffer, then were denatured (10 min) at 70°C and re-annealed (30 min) at 37°C. The signal of rhodamine-labelled Dux was amplified with rabbit anti-rhodamine antibodies (2h; 1:250; Invitrogen) and Alexa555 donkey antibodies raised against rabbit IgG (1h; 1:500; Invitrogen). Nuclei were stained with DAPI in in PBS, washed in PBS, before coverslips mounted with VectaShield, immediately before imaging, which was carried out on Olympus spinning disk confocal system, taking Z stacks every 250nm. Individual images were prepared for presentation (Figure 5A) using Max Projections (FIJI) of selected slices to show the location of *Dux* DNA loci.

Supplemental Materials and Methods contd

NAD RNA-seq analysis

For analysis of NAD gene expression, NAD regions defined in ESCs were taken from Bizhanova et al., 2020(Bizhanova et al. 2020). Type I NADs (overlapping constitutive LADs) and Type II NADs (overlapping constitutive interLADs) were defined as described in Vertii et al., 2019(Vertii et al. 2019) using LAD data from Peric-Hupkes et al., 2010(Peric-Hupkes et al. 2010), after LAD coordinates were shifted to mm10 using LiftOver tool (UCSC Genome Browser). For an example of NAD/LAD classification at the *Dux* locus see Figure S7. Ranked lists of $\log_2$ fold-change were prepared from RNA-seq of Pol I inhibitor treated ESCs (this study) and Ncl and LINE1 knockdown ESCs(Percharde et al. 2018). Ranked lists and Type I and Type II NAD files were submitted to the GSEA pre-ranked tool (genepattern.org) with the following parameters: permutations = 10000, collapse dataset = No_Collapse, and max gene set size = 4000. Normalised enrichment score (NES) and false discovery rate q-value (FDR) for each RNA-seq dataset were plotted using R/ggplot2.
